# An information-theoretic approach to the modeling and analysis of whole-genome bisulfite sequencing data

**DOI:** 10.1186/s12859-018-2086-5

**Published:** 2018-03-07

**Authors:** Garrett Jenkinson, Jordi Abante, Andrew P. Feinberg, John Goutsias

**Affiliations:** 1 Whitaker Biomedical Engineering Institute, Johns Hopkins University, Baltimore, MD, USA; 2 Center for Epigenetics, Johns Hopkins School of Medicine, Baltimore, MD, USA; 3 Department of Biomedical Engineering, Johns Hopkins University, Baltimore, MD, USA; 4 Department of Medicine, Johns Hopkins School of Medicine, Baltimore, MD, USA

**Keywords:** DNA methylation, Genome analysis, Information theory, Ising model, Methylation analysis, WGBS data modeling and analysis

## Abstract

**Background:**

DNA methylation is a stable form of epigenetic memory used by cells to control gene expression. Whole genome bisulfite sequencing (WGBS) has emerged as a gold-standard experimental technique for studying DNA methylation by producing high resolution genome-wide methylation profiles. Statistical modeling and analysis is employed to computationally extract and quantify information from these profiles in an effort to identify regions of the genome that demonstrate crucial or aberrant epigenetic behavior. However, the performance of most currently available methods for methylation analysis is hampered by their inability to directly account for statistical dependencies between neighboring methylation sites, thus ignoring significant information available in WGBS reads.

**Results:**

We present a powerful information-theoretic approach for genome-wide modeling and analysis of WGBS data based on the 1D Ising model of statistical physics. This approach takes into account correlations in methylation by utilizing a joint probability model that encapsulates all information available in WGBS methylation reads and produces accurate results even when applied on single WGBS samples with low coverage. Using the Shannon entropy, our approach provides a rigorous quantification of methylation stochasticity in individual WGBS samples genome-wide. Furthermore, it utilizes the Jensen-Shannon distance to evaluate differences in methylation distributions between a test and a reference sample. Differential performance assessment using simulated and real human lung normal/cancer data demonstrate a clear superiority of our approach over DSS, a recently proposed method for WGBS data analysis. Critically, these results demonstrate that marginal methods become statistically invalid when correlations are present in the data.

**Conclusions:**

This contribution demonstrates clear benefits and the necessity of modeling joint probability distributions of methylation using the 1D Ising model of statistical physics and of quantifying methylation stochasticity using concepts from information theory. By employing this methodology, substantial improvement of DNA methylation analysis can be achieved by effectively taking into account the massive amount of statistical information available in WGBS data, which is largely ignored by existing methods.

**Electronic supplementary material:**

The online version of this article (10.1186/s12859-018-2086-5) contains supplementary material, which is available to authorized users.

## Background

DNA methylation is a stable epigenetic mechanism that chemically marks the DNA by adding methyl (CH_3_) groups at individual cytosines immediately adjacent to guanines. Methylation marks are used to identify cell-type specific aspects of gene regulation, since marks located within a gene promoter or enhancer typically act to repress gene transcription, whereas promoter or enhancer demethylation is associated with gene activation. Notably, patterns of methylation marks are highly polymorphic and stochastic [1] containing information about a broad range of normal and aberrant biological processes, such as development and differentiation, aging, and carcinogenesis [2, 3].

Although several experimental assays have been designed to map DNA methylation marks, whole-genome bisulfite sequencing (WGBS) is increasingly becoming the method of choice due to its high quantitative accuracy, resolution, and genome-wide coverage [4]. Extraction of methylation information from bisulfite data has led to many parametric and non-parametric methods for modeling, analysis, and interpretation [4, 5]. Most methods, however, ignore correlations, an important aspect of methylation that has been observed within genomic regions of several CpG dinucleotides, at least over small distances [6–8]. Recent analysis methods for bisulfite sequencing data take into account correlation information indirectly by smoothing marginal statistics [9–16], or by *post hoc* corrections that empirically impose correlations among marginal statistics [17]. Other important methods follow a more direct approach, but they have only been designed to detect differential methylation in data obtained by Illumina’s 450k arrays [18, 19], whose continuous intensity measurements require fundamentally different models and methods, when compared to discrete sequencing reads.

It has been recently observed that fully characterizing the polymorphic and stochastic nature of DNA methylation requires specification of joint probability distributions of methylation patterns formed by sets of spatially coupled CpG sites [20, 21]. Motivated by this important observation, we recently introduced a DNA methylation model based on the 1D Ising distribution of statistical physics that directly takes into account correlations in methylation [22]. We showed that this model leads to a powerful approach to methylation analysis that allows a comprehensive genome-wide treatment of methylation stochasticity leading to a number of novel discoveries. By generating realistic synthetic data that take into account incomplete observations with given coverage (5-30 ×), and by computing median estimates and 95% confidence intervals for mean methylation levels and methylation entropies using extensive Monte Carlo simulations, we demonstrated in [22] that the empirical approach to joint methylation analysis used in [20] does not perform well when dealing with highly stochastic methylation data. Our Ising-based approach on the other hand results in exceptional statistical performance when estimating mean methylation levels and entropies, with their median values falling close to the true values and the 95% confidence intervals being relatively tight around the true values, even at low coverage.

Notably, an alternative statistical model has been recently proposed in [23] for the distribution of methylation patters at any given locus of the genome using a constrained multinomial model. However, this method is limited to methylation data with higher coverage than available in standard WGBS and results in modeling only a subset of the genome analyzed by techniques such as reduced representation bisulfite sequencing or captured assays. Moreover, this technique, as well as the methods proposed in [20, 21], cannot handle partial observations, leading to sparse modeling of the genome, and are subject to the *curse of dimensionality*, a problem associated with the exponential growth of model parameters that must be estimated from large (and most often forbidding) amounts of data. Furthermore, these techniques assign zero probabilities to unobserved methylation patterns despite their biological plausibility, which results in underestimating the true biological heterogeneity of methylation patterns [22].

In this paper, we focus on describing the algorithms that enable the 1D Ising model to be applied on WGBS data. We partition the genome into equally sized (in terms of bp’s) non-overlapping regions and use the Ising model to derive the probability mass function (PMF) of methylation within each genomic region, with each PMF specified by using only five parameters characteristic to the region. We then present iterative algorithms that compute and marginalize these PMFs, a crucial step for estimating the underlying parameters from WGBS data and for computing measures of methylation level, stochasticity and discordance. We subsequently discuss the problem of parameter estimation using maximum-likelihood and show identifiability of the parameters. We furthermore present methods for inter-sample and differential methylation analysis and develop novel schemes for classifying the methylation status in terms of methylation level and entropy throughout the genome. We also develop a new method for detecting differentially methylated regions (DMRs) using an information-theoretic measure of distance between two probability distributions, as well as a method for ranking epigenetically dysregulated genes in a test/reference study with or without replicates. Finally, by using simulated data, as well as three pairs of matched human lung normal/cancer WGBS samples, we show that our approach is superior when compared to DSS, a state-of-the-art method for genome-wide differential methylation analysis of WGBS data [15, 16]. Moreover, we provide clear evidence that metilene, a recently proposed method [24], cannot be reliably used for identifying aberrant methylation in a test/reference setting, since the statistical framework employed by this method is unable to attribute detected differential methylation activity to discordance in the test sample due to its high false positive rate. Further analysis of our lung data illustrates the effectiveness of our approach in producing information about the methylation status of the epigenome within different genomic features and at multiple scales, extracted from WGBS data in inter-sample or differential studies.

We refer to the proposed methodology as informME (**inform**ation-theoretic analysis of **ME**thylation), which we have implemented using MATLAB, C++, and R in a fully documented and publicly available software package that can be downloaded from GitHub (https://github.com/GarrettJenkinson/informME).

## Methods

### DNA methylation model

By following [22], we consider in this paper a genome comprising *N* CpG sites 1,2,…,*N*, which we label according to their order of appearance along the genome. Since the biochemical reactions that establish and maintain methylation are inherently stochastic, we represent the genome’s epigenetic state by an *N*×1 binary-valued random vector ***X*** whose *n*-th component *X*_*n*_ takes value *x*_*n*_=0, if the *n*-th CpG site is unmethylated, and value *x*_*n*_=1, if the site is methylated. We have argued in [22] that a natural choice for the PMF *P*_*X*_(***X***)= Pr[***X***= ***x***] of ***X*** is given by the 1D Ising model of statistical physics [25] with energy function $-\sum _{n=1}^{N} a_{n} (2x_{n}-1) - \! \sum _{n=2}^{N} \! c_{n}(2x_{n}-1)(2x_{n-1}-1)$. In this case,

1 $$ \begin{aligned} P_{X}(\boldsymbol{x}) &= \frac{1}{Z} \exp \left\{ \sum\limits_{n=1}^{N} a_{n} (2x_{n}-1) \right. \\ & \,\,\,\quad\qquad \left. + \sum\limits_{n=2}^{N} {c}_{n} (2x_{n}-1)(2x_{n-1}-1) \right\}, \end{aligned}  $$

where

2 $$ \begin{aligned} Z &= \sum\limits_{\boldsymbol{u}} \exp \! \left\{\sum\limits_{n=1}^{N} a_{n} (2u_{n}-1) \right. \\ & \!\qquad\qquad \left. + \sum_{n=2}^{N} c_{n} (2u_{n}-1) (2u_{n-1}-1) \right\} \end{aligned}  $$

is a constant known as the partition function. This model is expressed in terms of the location-dependent parameters *a*_*n*_ and *c*_*n*_, with *a*_*n*_ accounting for intrinsic factors that affect methylation at the *n*-th CpG site and *c*_*n*_ accounting for methylation cooperativity between the CpG sites *n*−1 and *n*. Notably, if *c*_*n*_=0 for all *n*, then the previous Ising model characterizes statistically independent methylation. Moreover, if *a*_*n*_=*a* and *c*_*n*_=*c* for all *n* (i.e., if the Ising parameters do not depend on location), then we can show that, when *a*<0 and *c*≥0, the most likely methylation state will be the fully unmethylated state, whereas, when *a*>0 and *c*≥0, the most likely state will be the fully methylated state. Finally, when *a*=0 and *c*>0, the most likely methylation state will be either the fully unmethylated or the fully methylated state, a behavior that is associated to methylation bistability.

The Ising model in (1) and (2) provides a joint PMF that fully encapsulates the methylation state of all CpG sites in the genome and represents a fundamentally different modeling paradigm from traditional tools that focus on marginally modeling one CpG site at a time. InformME is based upon leveraging the higher-order statistical information contained in the Ising model to provide information-theoretic quantities and insights that are fundamentally unavailable to marginal modeling methods or to methods that empirically estimate the joint PMF of methylation of a few CpG sites.

To compute the probability *P*_*X*_(***x***) of a methylation state ***X***, we need to estimate the 2*N*−1 parameters *a*_*n*_ and *c*_*n*_ from WGBS data, which is a prohibitively large number of parameters for reliable estimation. We address this problem by partitioning the genome into relatively small and equally sized (in terms of bp’s) non-overlapping regions ${\mathcal {R}}_{1},{\mathcal {R}}_{2},\ldots $, and by setting

3 $$ a_{n} = \alpha_{k} + \beta_{k} \rho_{n},   $$

and

4 $$ c_{n} = \frac{\gamma_k}{d_n},   $$

within each region ${\mathcal {R}}_{k}$, where *α*_*k*_, *β*_*k*_ and *γ*_*k*_ are three parameters characteristic to the genomic region, *ρ*_*n*_ is the CpG density at the *n*-th CpG site, given by

5 $$ {}\begin{aligned} \rho_{n} = \frac{1}{1000} \times &\left[ \text{\# of CpG sites within } \pm 500 \text{ nucleotides } \right. \\ &~~~~~\left.\text{ downstream and upstream of } {n} \right],  \end{aligned}  $$

and *d*_*n*_ is the distance of the *n*-th CpG site from its nearest-neighbor CpG site *n*−1, given by

6 $$ {}\begin{aligned} d_{n} & = \left[ \text{\# of bp steps along the DNA between the } \right. \\ & \left. ~~~~~~~\text{ cytosines of CpG sites } {n} \text{ and } n-1 \right]. \end{aligned}  $$

Note that (3) and (4) express the location-dependent parameters *a*_*n*_ and *c*_*n*_ of the Ising model within the genomic region ${\mathcal {R}}_{k}$ in terms of three location-independent parameters, *α*_*k*_, *β*_*k*_, and *γ*_*k*_. Parameter *α*_*k*_ accounts for intrinsic factors that uniformly affect methylation over the entire region, whereas parameter *β*_*k*_ modulates the influence of the CpG density *ρ*_*n*_ on methylation, in agreement with known results [26, 27]. On the other hand, (4) accounts for the fact that, due to the known processivity of the DNMT enzymes [28–30], the methylation status of contiguous CpG sites is most often highly correlated, with the correlation between the methylation states of two consecutive CpG sites decaying as the distance *d*_*n*_ between these two sites increases [6, 7, 31].

It is important to point out that the PMF of the methylation state within a genomic region ${\mathcal {R}}_{k}$ can be approximately expressed in terms of a 1D Ising model as well (Additional file 1: Section 1). Moreover, its partition function can be evaluated by an efficient iterative algorithm that allows computation of the PMF *P*_*X*_(*x*_1_,*x*_2_,…,*x*_*R*_) of methylation within ${\mathcal {R}}_{k}$ (Additional file 1: Section 2). Finally, marginal PMFs can be efficiently evaluated within ${\mathcal {R}}_{k}$ (Additional file 1: Section 3).

### Parameter estimation

Our results in Additional file 1, Section 1, show that, within each genomic region ${\mathcal {R}}_{k}$, DNA methylation can be approximately modeled by a 1D Ising model that is expressed in terms of only five parameters ${\boldsymbol {\theta }}_{k} = \left (\alpha ^{\prime }_{k},\alpha _{k},\alpha ^{\prime \prime }_{k},\beta _{k},\gamma _{k}\right)$ characteristic to the region. To estimate ***θ***_*k*_ from available data, first note that WGBS does not always measure the methylation state at all CpG sites within a genomic region, thus frequently producing incomplete data. To address this issue, we obtain an estimate $\widehat {\boldsymbol {\theta }}_{k}$ of ***θ***_*k*_ by solving the following maximum-likelihood estimation problem:

7 $$ \widehat{\boldsymbol{\theta}}_{k} = \arg\max_{{\boldsymbol{\theta}}_k} {\mathcal{L}}({\boldsymbol{\theta}}_{k}),   $$

where

8 $$ \mathcal{L}({\boldsymbol{\theta}}_{k}) = \frac{1}{M} \! \sum\limits_{m=1}^{M} \ln \! \left[ P_{\! X}\left(\left\{x_{r}^{(m)}, r \in {\mathcal{R}}_{k}(m)\right\} \left|\vphantom{x_{r}^{(m)}}\ {\boldsymbol{\theta}}_{k}\right) \right]\right.   $$

is the average “marginalized” log-likelihood function of ***θ***_*k*_ given *M* independent observations ***x***^(1)^,***x***^(2)^,…,***x***^(*M*)^ of the methylation state within the genomic region ${\mathcal {R}}_{k}$. In (8), ${\mathcal {R}}_{k}(m)$ is the set of all CpG sites within the genomic region ${\mathcal {R}}_{k}$ whose methylation state is measured in the *m*-th observation, and $P_{\! X}(\{x_{r}^{(m)}, r \in {\mathcal {R}}_{k}(m) \} \mid {\boldsymbol {\theta }}_{k})$ is the likelihood of the *m*-th observed sample obtained by marginalizing the entire likelihood *P*_*X*_(***x***∣***θ***_*k*_) over the “unmeasured” CpG sites.

Notably, we can show that the parameter vector ***θ***_*k*_ is *identifiable* (Additional file 1: Section 4). This implies that, for any two parameter vectors ${\boldsymbol {\theta }}^{\prime }_{k}$ and ${\boldsymbol {\theta }}^{\prime \prime }_{k}$ such that ${\boldsymbol {\theta }}^{\prime }_{k} \neq {\boldsymbol {\theta }}^{\prime \prime }_{k}$, we have $P_{\! X}(\boldsymbol {x} \mid {\boldsymbol {\theta }}^{\prime }_{k}) \neq P_{\!X}(\boldsymbol {x} \mid {\boldsymbol {\theta }}^{\prime \prime }_{k})$ for some ***x***. A non-identifiable parametrization can be problematic in statistical estimation, since it is possible in this case for two parameter values to be indistinguishable even when infinite data is available.

Calculating a marginal likelihood is computationally expensive if not intractable. However, when ${\mathcal {R}}_{k}(m)$ contains one contiguous set of CpG sites (which is most often the case with WGBS), we can compute the marginal likelihood exactly using the method discussed in Additional file 1, Section 3. On the other hand, when ${\mathcal {R}}_{k}(m)$ does not contain one contiguous set of CpG sites, we can compute the marginal likelihood approximately by partitioning ${\mathcal {R}}_{k}(m)$ into subsets of contiguous CpG sites, by calculating the marginal probability distributions over each subset, and by forming their product.

To strike a balance between computational and estimation performance, we empirically determined that a good choice for the length of each genomic region ${\mathcal {R}}_{k}$ used for parameter estimation is 3-kb. In addition, we choose not to model genomic regions that either have less than 10 CpG sites [because of concerns regarding statistical overfitting, as it would have to estimate 5 parameters from a small number (< 10) of variates], or for which there was insufficient data (less than 2/3 of the CpG sites were observed or the average depth of coverage for the region was less than 2.5 observations per CpG site). While this means that CpG sites in very low density genomic regions ${\mathcal {R}}_{k}$ will not be considered by informME, the vast majority of CpG sites can be modeled (99% of CpG sites in hg19). If desired, the remaining CpG sites could be modeled by traditional marginal methods, since correlations between very sparse CpG sites are expected to be negligible. Such modeling is commonly done by using Bismark’s methylation extractor tool and independent binomial models at each CpG site. Bismark is already used in the standard informME pipeline workflow to generate BAM files and, therefore, it is simple for a user of informME to model CpG sites in very low density regions if desired. Finally, in regions with sufficient data, we perform optimization using multilevel coordinate search [32], a global non-convex derivative-free strategy that outperforms other algorithms we considered (e.g., simulated annealing), in agreement with recent findings [33].

We determined the length of each genomic region ${\mathcal {R}}_{k}$ by employing low coverage data (7-10 ×) and by evaluating the previous maximum-likelihood estimation method in terms of estimation performance and computational efficiency with increasing region size (ranging from 1-kb to 10-kb). Overall, computational performance and overfitting became a concern for region sizes below 3-kb, leading to an appreciable number of genomic regions not being modeled by the estimation method, whereas, no noticeable loss in estimation performance was observed at region sizes above 3-kb. For better resolution, we therefore decided to use genomic regions with the smallest acceptable length of 3-kb. Note, however, that the size of each genomic region ${\mathcal {R}}_{k}$ employed for estimation is a parameter that users can set to their liking by employing any method of choice, such as a method based on Akaike’s information criterion (AIC) [34].

### Single-sample methylation analysis

#### Resolution

For high-resolution methylation analysis, we must consider genomic regions that are much smaller than the 3-kb regions ${\mathcal {R}}_{k}$ used for parameter estimation but large enough to account for correlations in methylation. Inspired by the length (about 146 bp) of the DNA within a nucleosome [35], we choose to partition each region ${\mathcal {R}}_{k}$ of the genome into genomic units (GUs) of 150 bp each and perform methylation analysis at a resolution of one GU. In humans, the number of CpG sites contained in each GU ranges from 0 to 44 (Additional file 2: Table S1).

Our statistical estimation can (approximately) provide the joint PMF of methylation within any genomic region of interest (by combining Ising probability distributions over consecutive estimation regions and by marginalizing the resulting PMF). As a consequence, informME can in theory be modified to include any desired definition of GUs, including non-uniformly or adaptively sized GUs, since the algorithms discussed in this paper are general enough to handle such cases. For simplicity and computational efficiency, however, we here consider uniformly sized GUs. We chose their size (150 bp) to be large enough in order to capture cooperativity among closely clustered CpG sites and small enough in order to perform methylation analysis at high resolution. informME allows users to modify the size of the GUs but it does not allow for non-uniformly or adaptively sized GUs at this time, although this could be implemented if desired without changing the underlying algorithms.

#### Methylation level

To characterize methylation within a GU containing *K* CpG sites *k*=1,2,…,*K* (labeled according to their order of appearance along the GU), we employ the methylation level

9 $$ L = \frac{1}{K}\sum_{k=1}^{K} X_{k}.   $$

Its PMF *P*_*L*_(*ℓ*)= Pr[*L*=*ℓ*], *ℓ*=0,1/*K*,…,1, satisfies

10 $$ P_{L}(\ell) = \sum_{{\boldsymbol{X}} \in {\mathcal{X}}(K \ell)} \Pr[{\boldsymbol{X}} = {\boldsymbol{x}}],   $$

where ${\mathcal {X}}(k)$ is the set of all methylation states within the GU with exactly *k* CpG sites being methylated. We calculate this PMF by using the method described in Additional file 1: Section 5.

#### Mean methylation level

To quantify methylation within a GU in a manner that is consistent with existing methods, we compute the mean methylation level (MML), given by

11 $$ {\mathrm{E}}[L] = \frac{1}{K} \sum_{k=1}^{K} {\mathrm{E}}[X_{k}] = \frac{1}{K} \sum_{k=1}^{K} \Pr[X_{k}=1].   $$

This is done genome-wide by calculating the probabilities Pr[*X*_*k*_=1] from the Ising model using the marginalization method discussed in Additional file 1: Section 3.

#### Methylation entropy

Methylation stochasticity is commonly quantified by computing means and variances at individual CpG sites. Due however to the complicated nature of the underlying probability distributions, a proper treatment requires use of higher-order statistics [18, 20, 22]. As such, the notion of epipolymorphism has been proposed as a joint measure of stochasticity [20]. However, previous analysis has demonstrated that this measure is generally not available methylome-wide and can dramatically underestimate heterogeneity, especially in the relatively low coverage data common to WGBS experiments [22]. We therefore choose to quantify methylation stochasticity within a GU comprised *N* CpG sites using a *normalized* version of the Shannon entropy, given by

12 $$ h = - \frac{1}{\log_{2}(N+1)} \sum_{\ell} P_{\! L}(\ell) \log_{2} \! P_{\! L}(\ell),   $$

which we refer to as the normalized methylation entropy (NME). This quantity takes values between 0 and 1, with larger values indicating higher levels of randomness in methylation level. Note that normalization allows comparison of methylation randomness within GUs containing different numbers of CpG sites, which otherwise would not be possible. For example, perfectly random methylation levels within two GUs with different numbers of CpG sites, *N*_1_ and *N*_2_, are characterized by the same NME value of 1, despite the fact that the GUs are associated with different Shannon entropies log2(*N*_1_+1) and log2(*N*_2_+1).

#### Classification of genomic units

**Fig. 1:**
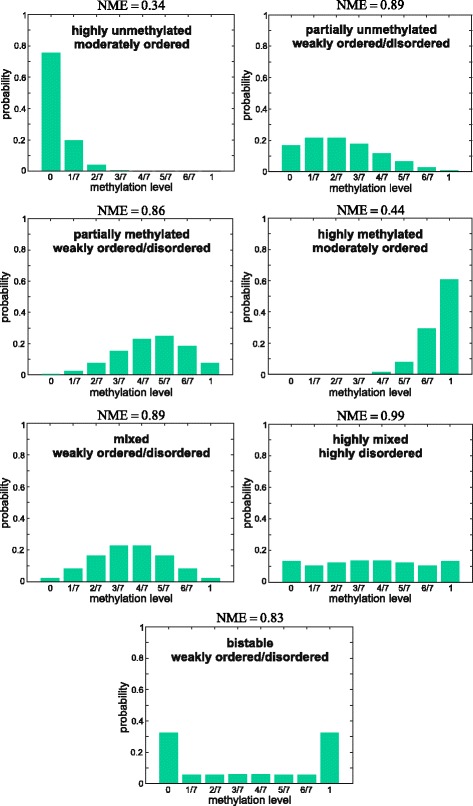
Examples of methylation level and entropy based classification of a GU that contains 7 CpG sites. The methylation based GU classification is determined by the shape of the methylation level PMF using the scheme described in Additional file 1, Section 6.1, whereas the entropy based GU classification is determined by the NME value using the scheme described in Additional file 1, Section 6.2

To provide an effective interpretation of the MML output, we developed a classification scheme that summarizes the status of methylation level within a GU based on the shape of its PMF (Additional file 1: Section 6.1). This scheme classifies a GU into one of seven classes: highly unmethylated, partially unmethylated, partially methylated, and highly methylated, as well as mixed, highly mixed, and bistable; see Fig. 1 for examples. In this scheme, mixed and highly mixed GUs are characterized by appreciable methylation variability. Moreover, bistable GUs are characterized by the highest possible variance in methylation level (Additional file 1: Section 6.1 and [36]), even higher than the variance associated with a highly mixed GU, and have been linked to gene imprinting [22].

By employing a simple thresholding scheme, we also classify a GU in terms of its entropy content into one of five categories (Additional file 1: Section 6.2): highly ordered, moderately ordered, weakly ordered/disordered, moderately disordered, and highly disordered; see Fig. 1 for examples. Highly ordered GUs are characterized by low variability of methylation level in a cell population, whereas highly disorder GUs are associated with areas of the genome that are subject to significant methylation randomness.

### Differential methylation analysis

#### Differential methylation level

To capture differences in methylation level within a GU between a test and a reference sample, we employ the random variable *D*_*L*_=*L*_*t*_−*L*_*r*_, where *L*_*t*_ and *L*_*r*_ are the methylation levels in the test and the reference sample, respectively. We can then evaluate differences in methylation level by calculating the differential mean methylation level (dMML) E[*D*_*L*_]=E[*L*_*t*_]−E[*L*_*r*_]. This is a measure of methylation dissimilarity that has been extensively used by existing methods for methylation analysis.

#### Classification of GUs

**Fig. 2:**
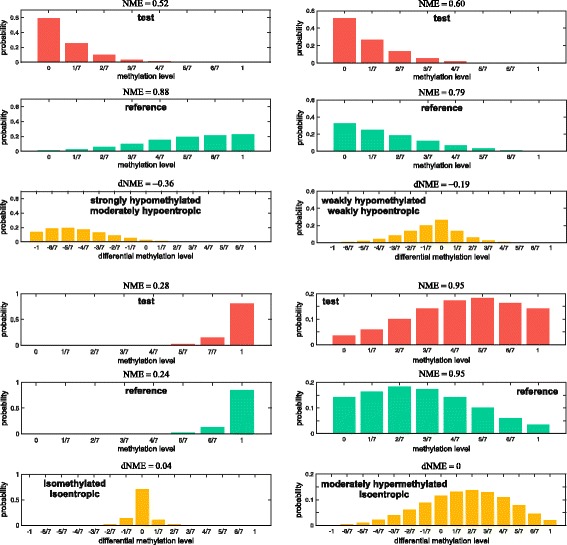
Examples of differential methylation level and entropy based classification of a GU that contains 7 CpG sites. The methylation based GU classification is determined by the shape of the PMF of the differential methylation level using the scheme described in Additional file 1, Section 7.1, whereas the entropy based GU classification is determined by the differential NME value using the scheme described in Additional file 1, Section 7.2

More generally, we calculate the PMF of *D*_*L*_ by convolving the PMFs of *L*_*t*_ and *L*_*r*_ (assuming that *L*_*t*_ and *L*_*r*_ are statistically independent). We then use the resulting PMF to interpret differences in methylation level using a scheme that classifies a GU into one of seven categories (Additional file 1: Section 7.1): strongly hypomethylated, moderately hypomethylated, weakly hypomethylated, isomethylated, weakly hypermethylated, moderately hypermethylated, and strongly hypermethylated; see Fig. 2 for examples.

#### Differential entropy

To capture entropy differences between a reference and a test sample, we compute the differential normalized methylation entropy (dNME) *D*_*h*_=*h*_*t*_−*h*_*r*_, where *h*_*t*_ and *h*_*r*_ are the NMEs within each sample. Moreover, by using a simple thresholding scheme, we classify each GU into one of seven classes (Additional file 1: Section 7.2): strongly hypoentropic, moderately hypoentropic, weakly hypoentropic, isoentropic, weakly hyperentropic, moderately hyperentropic, and strongly hyperentropic; see Fig. 2 for examples.

#### Differential probability distribution

Differential methylation analysis between two samples can also be performed by quantifying the dissimilarity between the PMFs $P^{(1)}_{\! L}$ and $P^{(2)}_{\! L}$ of the methylation levels within a GU using their Jensen-Shannon distance (JSD), given by [37]

13 $$ d = \sqrt{\frac{D \left(P^{(1)}_{\! L}, \overline{P}_{\! L}\right) + D \left(P^{(2)}_{\! L},\overline{P}_{\! L}\right)}{2}},   $$

where $\overline {P}_{\! L}(\ell) = \left [P^{(1)}_{\! L}(\ell)+P^{(2)}_{\! L}(\ell) \right ]\!/2$ is the average of the two PMFs and

14 $$ D(P,Q) = \sum_{\ell} P(\ell) \log_{2} \!\! \left [ \frac{P(\ell)} {Q(\ell)} \right ]   $$

is the Kullback-Leibler divergence between two probability distributions *P* and *Q*. It turns out that the JSD is a normalized metric, since it takes values between 0 and 1, it becomes zero if and only if $P^{(1)}_{\! L} = P^{(2)}_{\! L}$, it is symmetric, and satisfies the triangle inequality [38]. Moreover, it reaches its maximum value of 1 if the supports of the two PMFs do not intersect each other, in which case the PMFs can be perfectly distinguished from a single sample.

**Fig. 3:**
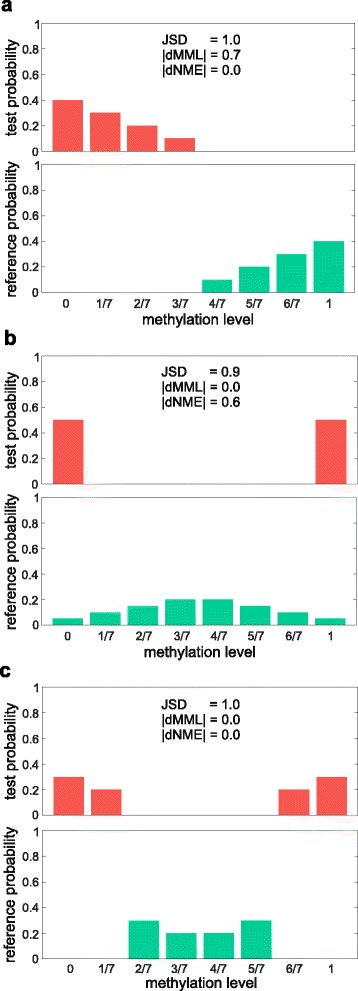
Examples of methylation level PMFs within a GU containing 7 CpG sites with a high JSD value between a test and a reference sample: **a** The observed high JSD value of 1 is mainly driven by a high absolute dMML of 0.7. **b** The high JSD value of 0.9 is mainly driven by a high absolute dNME of 0.6. **c** A high JSD value can be due to statistical factors other than a nonzero dMML or a nonzero dNME. The depicted PMFs result in the highest JSD value of 1, despite the fact that they result in zero dMML and dNME values

It is important to note here that a high JSD value may be driven by a difference in MML, NME or both, or by other statistical factors that are not accounted for by the mean or entropy; see Fig. 3. This implies that using the JSD as a dissimilarity measure for detecting crucial or aberrant differences in the stochastic behavior of DNA methylation may lead to biological findings that are concealed from observation when employing traditional differential methylation analysis methods based on mean methylation or even entropy differences. We illustrate this crucial point in the next section by analyzing WGBS data associated with lung normal/cancer samples.

#### DMR detection

An objective of WGBS data analysis is to detect DMRs; i.e., stretches of DNA in which appreciable differences in methylation are observed. Here, we discuss a novel algorithm that defines a DMR as a region of the genome that exhibits statistically significant differences in the PMFs of methylation level between a test and a reference sample, as quantified by the JSD. As a consequence, this approach can account for non-mean based differences that would otherwise be missed by existing methods designed to detect DMRs in WGBS data.

The most biologically relevant changes in methylation are expected to occur in GUs with high JSD values and across regions containing many such GUs. Our approach, however, computes JSD values within GUs independently, leading to a signal that can change rapidly from one GU to the next. To address this issue, we compute *smoothed* JSD (sJSD) values by applying the Nadaraya-Watson kernel regression smoother with a Gaussian kernel of fixed bandwidth (which controls the scale of the DMR finder) on the original JSD values. This is implemented by using the R function ksmooth with a bandwidth of 50-kb, corresponding to a kernel with standard deviation of about 18.5-kb, which was found to be effective in most cases.

When replicate reference data is available, we first evaluate the genome-wide empirical *null* distribution of all observed sJSD values between pairs of replicate reference WGBS samples. Given the sJSD value within a GU computed from a test/reference sample, we then calculate the probability (*p*-value) that, by chance, the sJSD is at least as large as the observed value due to biological, statistical, and technical variability in the reference samples. Subsequently, we perform multiple hypothesis testing using the Benjamini-Yekutieli (BY) method [39] for controlling the false discovery rate (FDR) at 0.01, which leads to a maximum of 1% of the GUs identified by our method to be false positives on the average. The BY procedure is a conservative modification of the original Benjamini-Hochberg (BH) method [40] and has been shown to control the FDR for dependent test statistics. Note, however, that our JSD-based DMR algorithm can also be implemented using the BH procedure, which was shown to control the FDR in the particular type of positive regression dependency [39], or using any other FDR control procedure of choice. Finally, we convert the *q*-value associated with a differentially methylated GU to a statistical quality score (SQS), given by SQS=−10log_10_(*q*), and use this measure to quantify the statistical significance of the GU.

The union of all GUs identified by the previous method form a set of DMRs that are sparse due to independent analysis. To reduce sparsity, we fill-in gaps between neighboring DMRs of size smaller than the sJSD smoothing bandwidth (taken to be 50-kb) by applying a morphological closing [41] on the binary signal of DMR classification. Moreover, we annotate each resulting connected DMR by a statistical score, which we compute by summing all SQS values within the DMR. This allows ranking of the DMRs based on the amount of statistical evidence within each region.

When replicate reference data is not available, we compute the null distribution of sJSD values from a single pair of test/reference samples by assuming that the sJSD value within a randomly selected GU is associated with (*i*) a difference in the methylation level PMFs within the GU that is only due to biological, statistical and technical variability (null hypothesis), or (*ii*) a difference that is also due to distinct epigenetic behavior (alternative hypothesis). In this case, we can model the genome-wide distribution of appropriately transformed sJSD values (to be between −*∞* and *∞*) using a Gaussian mixture model comprising two components: one that corresponds to case (*i*) and one that corresponds to case (*ii*). The Gaussian component corresponding to case (*i*) can then be used to model and compute the desired null distribution.

To build this mixture model, we transform the sJSD values using the logit function

$$ \text{logit}(x) = \log \! \left (\frac{x}{1-x} \right).    $$

**Fig. 4:**
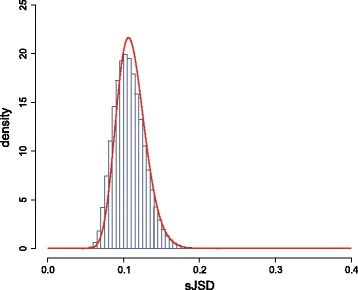
Genome-wide empirical distribution of all sJSD values, obtained by comparing three lung normal samples (blue). This distribution can be well approximated by a logit-normal distribution (red)

We then employ the R package mixtools to estimate a mixture of two Gaussian distributions that best fits the empirical distribution of the observed logit-transformed sJSD values using the EM algorithm. This produces the means *μ*_1_, *μ*_2_ and variances *σ*_1_, *σ*_2_ of the two Gaussian distributions, as well as the corresponding weights *w*_1_ and *w*_2_. We expect that, on the average, the sJSD values in case (*i*) will be smaller than the sJSD values in case (*ii*). This leads us to expect that the null distribution of the logit-transformed sJSD values can be well approximated by the Gaussian mixture component associated with the smallest mean value. As a result, we can approximate the null distribution of the sJSD values using the logit-normal distribution

$$ f(x) = \frac{1}{\sigma \sqrt{2 \pi}} \frac{1}{x(1-x)} \exp \left \{ - \frac{[\text{logit}(x) - \mu]^{2}} {2\sigma^{2}} \right \},    $$

where *μ*= min{*μ*_1_,*μ*_2_} and *σ* is the standard deviation of the Gaussian mixture component with mean *μ*. We demonstrate the validity of this approach in Fig. 4.

We expect that, on the average, sJSD values associated only with biological, statistical, and technical variability to be smaller than sJSD values associated only with distinct epigenetic behavior. This allows us to use the Gaussian component of the previously computed mixture with the smallest mean value as a model for the null distribution of the logit-transformed sJSD values. As a consequence, we approximately compute the null distribution of actual sJSD values from a single pair of test/reference samples using a logit-normal distribution and employ this distribution to perform hypothesis testing using the same method as the one employed when replicate reference data is available.

#### Ranking epigenetically dysregulated genes

DMR analysis is feature agnostic and genome-wide, making it possible to effectively focus on regions of the genome that exhibit most significant differences in methylation. If however the focus of analysis is more limited in scope, such as identifying genes subject to differential methylation, then DMR analysis will not be appropriate. Instead, one should limit statistical analysis to only features of interest (e.g., ranking gene promoters). This is due to the fact that a more targeted analysis will result in higher statistical power when detecting methylation differences at finer scales.

In this paper, we rank epigenetically dysregulated genes by determining, for each primary transcript in the human genome (possibly multiple per gene), its promoter region. We do this by identifying its transcription start site (TSS) and by centering a 4-kb window at that site. When reference replicate data are not available, we score a promoter region by the average JSD values of all GUs that intersect the region and use these scores to rank all promoters, with a higher score indicating a promoter that exhibits stronger differential methylation.

When replicate reference data is available, we rank a promoter region by following three steps. For each GU in the genome, we first test the null hypothesis that an observed dissimilarity in the PMFs of the methylation levels within the GU is due to biological, statistical, and technical variability against the alternative hypothesis that it is not. To implement this test, we use the JSD as the test statistic and construct an “empirical” null model [42] by approximating the genome-wide distribution of the JSD under the null hypothesis using the empirical distribution of the observed JSD values between all pairs of available replicate reference samples. Given the JSD value within a GU computed from a test/reference sample, we then calculate the probability (*p*-value) that, by chance, the JSD can be at least as large as the observed value due to biological, statistical, and technical variability in the reference samples. Subsequently, and for each promoter region, we combine the computed *p*-values of all GUs that intersect the region using Fisher’s method [43], score them using the resulting combined *p*-values, and use these scores to rank all promoters, with a lower score indicating a promoter that exhibits higher differential methylation. Note that the combined *p*-values are only exact when methylation within GUs is mutually independent, which is not in general true. However, we can still use the Fisher-based *p*-values as scores to effectively *rank* the promoter regions.

Finally, we obtain the desired list of ranked genes by associating promoter regions with their corresponding genes (possibly multiple promoters per gene) and by keeping only the highest ranking of a gene.

## Results

### WGBS data samples

To illustrate the appropriateness of informME and its superiority for methylation analysis over recently proposed methods, we used WGBS data corresponding to three pairs of *matched* lung normal/cancer samples: lungnormal-1 (14×), lungcancer-1 (15×), lungnormal-2 (10×), lungcancer-2 (10×), lungnormal-3 (19×), and lungcancer-3 (18×), where the numbers in parentheses indicate average genome-wide coverage. The sequencing data and the modeling results can be obtained from NCBI’s Gene Expression Omnibus (https://www.ncbi.nlm.nih.gov/geo), SuperSeries number GSE86340 (accession numbers GSM2103014-19).

### **Model evaluation**

We evaluated the appropriateness of modeling WGBS data using the Ising model $P^{(1)}_{\! X}$ in (1) and (2) with parameters that satisfy (3) and (4) to the more general Ising model $P^{(2)}_{\! X}$ whose parameters do not satisfy (3) and (4). We did so by randomly selecting, through the entire genome, a total of 10,000 3-kb estimation regions ${\mathcal {R}}_{k}$ modeled by informME in lungnormal-2, by fitting the two models within each region, and by computing Akaike’s information criterion (AIC), given by [34]

15 $$ {\begin{aligned} {}\text{AIC}_{i}(k) &= -2 \sum\limits_{m=1}^{M(k)} \! \ln {\left [ P^{(i)}_{\! X}\left(\left\{x_{r}^{(m)} \!, r \in {\mathcal{R}}_{k}(m) \right\}\!\! \mid \widehat{{\boldsymbol{\theta}}}_{i}(k)\right) \right ]}\\ &\quad + 2p_{i}(k), \end{aligned}}  $$

for *i*=1,2. In this equation, *M*(*k*) is the number of available observations within an estimation region ${\mathcal {R}}_{k}$, ${\mathcal {R}}_{k}(m)$ is the set of all CpG sites within ${\mathcal {R}}_{k}$ whose methylation state is measured in the *m*-th observation, $P^{(i)}_{\! X}(\{x_{r}^{(m)}, r \in {\mathcal {R}}_{k}(m) \} \mid {\boldsymbol {\theta }})$ is the likelihood of the *m*-th observed sample associated with the *i*-th model, obtained by marginalizing the entire likelihood $P^{(i)}_{\! X}({\boldsymbol {x}} \mid {\boldsymbol {\theta }})$ over the “unmeasured” CpG sites, $\widehat {{\boldsymbol {\theta }}}_{i}(k)$ is the maximum-likelihood estimate of the parameters associated with the *i*-th model, and *p*_*i*_(*k*) is the corresponding number of free parameters [ *p*_1_(*k*)=5 and *p*_2_(*k*)=2*R*(*k*)−1, with *R*(*k*) being the number of CpG sites in ${\mathcal {R}}_{k}$]. We then calculated the AIC probability *π*(*k*) that the Ising model with parameters that satisfy (3) and (4) is the best model for the data. This probability is given by [34]

16 $$ \pi(k) = \frac{\exp \{ -\Delta_{1}(k)/2\}} {\exp\{-\Delta_{1}(k)/2\} + \exp\{-\Delta_{2}(k)/2\}},   $$

where *Δ*_*i*_(*k*)=AIC_*i*_(*k*)− min{AIC_1_(*k*),AIC_2_(*k*)}, for *i*=1,2.

We found that 98% of the selected regions had AIC probability larger than 0.99 in favor of the simpler model, thus validating its superiority over the general Ising model for the particular WGBS data used. We expect this to be the case in practice, since very high coverage is required to support the more complex model, which would generally be prohibitively expensive using current WGBS technology.

### Differential performance assessment using simulated data

We also sought to investigate the differential performance of informME as compared to other methods for methylation analysis of WGBS data published in the literature. Existing methods for differential WGBS analysis are theoretically similar to each other in that they use marginal statistics, possibly in conjunction with a smoothing function, to statistically determine methylation differences at individual CpG sites. One such recent method, known as DSS [15, 16], has been compared to several methods (such as methylKit [9], BSmooth [10], BiSeq [11], RADMeth [12], and MOABS [14]), using simulated as well as real data and has been found to be more preferable than these methods. Moreover, metilene, a recently proposed DMR finder [24], was found to be superior to BSmooth and MOABS in terms of sensitivity (true positive rate), specificity (true negative rate), and speed of implementation on simulated data. However, our analysis in the next subsection and in the Additional file 1, Section 8, clearly demonstrates that DSS is statistically superior to metilene, since the latter method cannot produce differential methylation results that can be considered valid from a statistical perspective. For this reason, we chose to compare the differential performance of informME only to that of DSS.

We did so by first using the Ising model to generate synthetic methylation data that imitate the structure of the real samples we use in this paper (i.e., we generated three matched pairs of test and reference samples). Our synthetic samples behave like real sequencing data, with reads placed randomly along the genome. This means that the coverage of the CpG sites varies randomly along the DNA and that each read covers only a small fraction of the genome. We considered reads of 300 bp long and generated synthetic data with an average genome-wide coverage of 15×, which is common in WGBS. For simplicity, we modeled a synthetic genome having 5000 isolated CpG islands (CGIs) separated by gaps of 100-kb, with each CGI being 3-kb long and containing 200 uniformly spaced CpG sites.

Because CpG sites within each CGI are uniformly spaced, the Ising model is reduced to a two-parameter model (i.e., an Ising model with parameters *a* and *c* within each estimation region). For both test and reference samples, we set *a*=0. However, to impart a difference in the correlation between the two cases, we set *c*=0 in the test samples and *c*=*δ* in the reference samples, with *δ*=0.4,0.6,…,2.0. We did not include biological variability in the model, since our goal here is to simply show that marginal methods, such as DSS, cannot detect high-order differences in the joint probability distributions of methylation. Note also that, in this setup, the true marginal methylation means are identical (i.e., every CpG site has a true probability of 0.5 to be methylated in the test and the reference samples). We therefore expect that a marginal method of analysis, such as DSS, will not detect differential activity when using our synthetic samples. We also expect the sensitivity (true positive rate) of DSS to be equal to the Type I error rate (false positive rate), indicating a performance that is no better than random guessing.

**Fig. 5:**
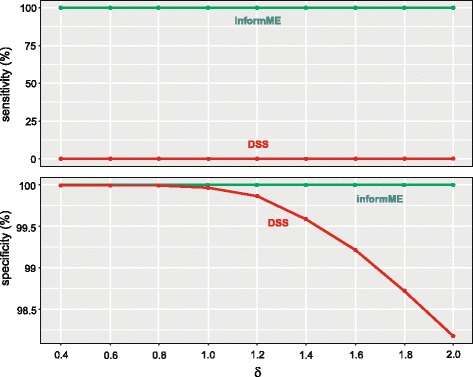
Sensitivity and specificity of informME and DSS when applied on simulated data based on three test/reference comparisons (for sensitivity) and three reference/reference comparisons (for specificity) as a function of the difference *δ* between the *c* parameter values of the Ising model in the test and reference samples

When applied on our three test/reference comparisons, informME produced 100% sensitivity for all values of *δ*, whereas it consistently resulted in 100% specificity (true negative rate) when it was applied on our three reference/reference comparisons; see Fig. 5. In the test/reference comparisons, informME identified every single CpG site as being differentially methylated, whereas in the reference/reference comparisons, informME detected no DMRs. For this simulation, we employed the default settings of our JSD-based DMR algorithm, except that we used a bandwidth of 1-kb (instead of the default value of 50-kb) to indicate that the sizes of our features of interest are of the order of 1-kb. These results demonstrate the statistical validity of DMR detection using informME, which can appropriately handle variations in coverage encountered in practice without resulting in a large Type I error rate (which equals to 1−specificity), while retaining the ability to detect real methylation differences when present.

DSS produced near zero sensitivity for all values of *δ*, whereas its specificity monotonically decreased with increasing values of *δ*; see Fig. 5. We attribute the lack of sensitivity to the fact that DSS is unable to reliably detect differences between the joint probability distributions of methylation other than in the mean, even when these differences are large, which is the case in our simulations. Notably, the differences in the joint probability distributions considered here were so large that informME never failed to detect their presence. On the other hand, the observed decrease in specificity demonstrates that correlations can lead to DSS not properly controlling the Type I error rate (maximum rate observed in our simulations was 0.018), since it appreciably exceeded the *p*-value threshold used by DSS by two orders of magnitude (in our testing, we used DSS’s default threshold of 10^−5^).

The previous findings demonstrate that not only do marginal methods, such as DSS, fail to detect high-order differences in methylation when present, but also that their statistical testing framework can become invalid due to their inability to model correlations in the data. In particular, we found that DSS, being based on a well-formed hypothesis testing framework, was able to control the Type I error rate in our reference/reference comparisons when there were small correlations and no biological variability. However, in the presence of larger correlations, DSS can lead to a Type I error rate that is many orders of magnitude higher than the chosen level (*p*-value threshold) used to control this error rate. This shows that, even when we are not concerned with detecting non-mean based differences in methylation, we must still utilize a modeling tool, such as informME, which properly accounts for correlations that are known to occur in real DNA methylation data.

### Differential performance assessment using real cancer/normal data

Assessing sensitivity and specificity of differential methylation analysis using simulated data favors methods that are compatible with the underlying theoretical assumptions pertaining to the models used for generating the data and can, therefore, lead to misleading conclusions. In addition, the practice in [15, 16] of evaluating methods based on the overlap of detected methylation differences with certain genomic features (such as gene promoters, CpG island shores, etc.) can be problematic since it requires prior division of the genome into regions of high versus low differential methylation activity, which is not possible in general. Finally, using real WGBS data to compare methods requires knowledge of ground truth information about the locations of differential methylation activity.

Statistical methods for identifying differential activity in a test/reference study are typically based on a hypothesis testing approach. Critically important to any hypothesis testing framework, however, is setting up a null hypothesis that is appropriate for the specific biological problem at hand. Since our interest here is to identify differential methylation in test versus reference samples (e.g., cancer versus normal) using WGBS data, we must test against the null hypothesis that observed differential activity is due to biological, statistical, or technical variability. Building a null model in this manner ensures that all sources of normal variability that might appear between a pair of reference samples are accounted for, whereas differences that exceed the norm under this null model can be assumed to be due to the test condition rather than other sources of variability (i.e., statistical sampling noise, technical noise from sequencing experiments, or normal biological variability in the reference tissue). By definition, if the null hypothesis is true, then the probability that a *p*-value is less than or equal to *α* will be *α* as well. This implies that the *p*-value will be uniformly distributed between 0 and 1. Thus, if we apply a differential methylation analysis method on our normal lung reference samples, we would expect a statistically sound hypothesis testing problem to produce, under the aforementioned null hypothesis (i.e., one that includes biological, technical and statistical variability), *p*-values whose genomewide empirical distribution is approximately uniform.

**Fig. 6:**
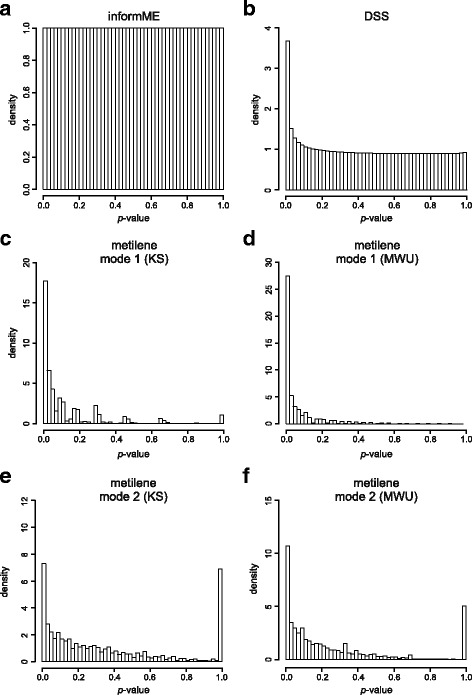
Distribution of *p*-values obtained genomewide using all three pairs of our lung normal data by: **a** informME, **b** DSS-single, **c** metilene in the “DMR de-novo annotation” mode 1 based on the KS test statistic, **d** metilene in the “DMR de-novo annotation” mode 1 based on the MWU test statistic, **e** metilene in “DMR annotation in known features” mode 2 based on the KS test statistic, and **f** metilene in “DMR annotation in known features” mode 2 based on the MWU test statistic

By applying informME on the three pairs of our lung normal data, we obtained *p*-values for each GU of the genome that follow a uniform empirical probability distribution; see Fig. 6a and Additional file 1: Figures S3-S5. However, when we applied DSS-single, we obtained the nonuniform empirical probability distribution depicted in Fig. 6b (see also Additional file 1: Figures S3-S5). We can view this probability distribution as a mixture of two components: a uniform null distribution attributed to statistical variability modeled by DSS-single and a nonuniform null distribution with additional probability mass concentrated over small *p*-values, which can be attributed to non-modeled biological or technical variability. We therefore conclude that DSS-single is not fully accounting for biological or technical variability in the data. Hence, differential methylation activity in a cancer/normal comparison detected by this algorithm cannot be necessarily attributed to cancer. However, Fig. 6(b) implies that, under the null hypothesis, the false positive rate of DSS-single due to biological or statistical variability (the area of the peak at 0) is relatively small (about 7.5%), as we would expect in a normal/normal comparison.

When we applied each of the two modes of metilene on our lung normal data [mode 1: DMR de-novo annotation; mode 2: DMR annotation in known features (promoters); see http://www.bioinf.uni-leipzig.de/Software/metilene], we obtained nonuniform empirical probability distributions for the *p*-values associated with the detected DMRs; see Figs. 6(c-f) and Additional file 1: Figures S3-S5. These *p*-values were obtained by using a 2D version of the Kolmogorov-Smirnov (KS) test or the Mann-Whitney U (MWU) test. In this case, it is not possible to view the resulting probability distributions as mixtures of two separate components. Moreover, the results show a much higher false detection rate than DSS under the null hypothesis (35% for KS mode 1, 55% for MWU mode 1, 15% for KS mode 2, and 20% for MWU mode 2) – see also Additional file 1: Section 8 for a theoretical discussion on why this is so. As a consequence, we do not believe that metilene can be reliably used for differential methylation analysis since it cannot statistically attribute detected differential methylation activity to cancer. Due to its unreasonably high false detection rate, a great deal of identified differential activity will be due to biological, statistical, or technical variability and not due to cancer.

A nonuniform probability distribution of *p*-values under the null hypothesis indicates that the test statistic used by a particular method for differential methylation analysis is not appropriate for testing against the previously articulated null hypothesis. DSS does a much better job than metilene in this respect, although informME is clearly the best method among the three to accomplish this goal. For this reason, we provide in the following a further assessment of the performance of informME and DSS when applied on real data.

We used gene ontology (GO) enrichment analysis (http://cbl-gorilla.cs.technion.ac.il) [44] to compare performance by evaluating the potential of informME to that of DSS for addressing a specific problem of interest to epigenetic biology: identifying biological processes that are significantly enriched in epigenetically dysregulated genes. By using GO enrichment analysis on gene lists of equal size formed by selecting genes with the largest detected methylation discordance at their promoters, we can remove the issue of sensitivity and specificity and focus on the ability of each method to produce biologically relevant results.

It is important to note that the gene selection method used in [16] selects a gene by checking whether a statistic *T*, which counts the number of the top 2000 differentially methylated CpG sites in the gene, is above a threshold *t*= 4. Unfortunately, this gene selection process produced no results in our data and, therefore, it cannot be reliably used to perform GO annotation.

The reason for this problem is that GO results depend on the size of the target list used (the set of selected genes), which must contain many genes, while the previous DSS-based selection process produces very few genes meeting the underlying criteria for selection. In our experience, to perform meaningful GO enrichment analysis, the target list should be about 1-3% the length of the background list (the set of all genes in the genome). Therefore, and to be fair when comparing DSS to informME, we sought to modify the gene selection process associated with DSS so that the two approaches select the same number of differentially methylated genes. We determined this number to be 450 genes so that the target list is approximately 2% of all genes (22,337 genes). Our modification consists of selecting a gene by thresholding a statistic *T*^′^ that counts the number of differentially methylated CpG sites in the gene (and not only the top 2000 sites), as determined by DSS, with a threshold that is adaptively chosen so that the target list contains 450 genes.

When using DSS, we can order genes by employing the *T*^′^ statistic discussed above. This implies that genes with more differentially methylated CpG sites within their promoters will be placed higher in the list. However, a major limitation of this procedure, which is not an issue with informME, is the fact that many genes will have no differentially methylated CpG sites in their promoters, as detected by DSS, resulting in many tied rankings at the bottom of the list. This can be detrimental to GO enrichment analysis using a single ranked list. Therefore, and in order to be fair to DSS, we focused on performing GO enrichment analysis using unranked target and background sets of genes for both informME and DSS, which require only a selection of 450 genes from the top of the ranked lists.

By adopting the previous strategy, we evaluated the performance of informME in the following three typical scenarios and found it to outperform DSS in producing the most biologically relevant outcomes.

#### Scenario 1 – Multiple pairs of matched test/reference samples are available

We applied informME on each pair of the matched cancer/normal samples in the lung dataset and, by using the fact that replicate reference data are available in this case, we ranked genes using our JSD-based Fisher approach (Additional file 2: Table S2). We then combined the results of the three comparisons into a single ranked list using the method of rank products [45, 46], implemented by the Bioconductor package RankProd, which provided a target list of 450 genes for GO analysis that are highly scored in all three comparisons. We also applied DSS-single on each pair of matched cancer/normal samples using the Bioconductor package DSS, ranked the genes based on the number of identified differentially methylated CpG sites within their promoters, and used rank products to combine the three ranked lists into a single list (Additional file 2: Table S3). This again provided a target list of 450 genes for GO analysis that are highly scored in all three comparisons.

informME identified many genes as being differentially methylated in lung cancer with several of them being discovered by DSS as well. Notably, 31 out of the top 50 genes identified by informME, such as *SALL3*, *HOXA5*, *SOX1*, *ZIC1*, *CBLN1*, *AJAP1*, *DIO3*, *GFRA1*, and *FOXC2*, have been already associated with lung cancer (Additional file 2: Table S4). Moreover, 19 out of the top 50 genes identified by informME were ranked among the top 100 differentially methylated genes by DSS. We noticed, however, that the rankings of some genes that are highly ranked by informME, such as *CBLN1*, *AJAP1*, *GFRA1*, and *FOXC2*, were substantially reduced by DSS.

We then employed GO enrichment analysis using a background set of 22,337 genes and a target set of the top 450 genes identified by each method. We limited the results to statistically significant GO terms (FDR *q*-value ≤ 0.05) that were also associated with at least 5 genes in the target set. The results, summarized in Table 1, show that informME produced 205 GO terms, with 38 of them having enrichment of at least 5. The highly enriched GO terms included many developmental and differentiation processes, such as patterning, regionalization, epithelial cell differentiation, and cell fate determination and commitment, as well as many cellular processes and corresponding pathways, such as cell communication, cell fusion, signalling, and chromatin silencing (Additional file 2: Table S5a). It also included processes associated with neurogenesis, as well as neuron fate specification, differentiation and commitment, which have been increasingly associated with lung and other types of cancer [47–49]. Notably, DSS produced an order of magnitude fewer GO terms (21 terms) with only 1 having enrichment of at least 5.

**Table 1:** Summary of GO enrichment analysis results when comparing informME to DSS

**SCENARIO 1**	**informME**	**DSS**
*lungcancer-VS-lungnormal*		
GO terms	205	21
GO terms (enrichment ≥ 5)	38	1
**SCENARIO 2**	**informME**	**DSS**
*lungcancer-VS-lungnormal*		
GO terms	167	3
GO terms (enrichment ≥ 5)	29	1
**SCENARIO 3**	**informME**	**DSS**
*lungcancer-1-VS-lungnormal-1*		
GO terms	176	68
GO terms (enrichment ≥ 5)	31	9
*lungcancer-2-VS-lungnormal-2*		
GO terms	148	2
GO terms (enrichment ≥ 5)	25	0
*lungcancer-3-VS-lungnormal-3*		
GO terms	159	42
GO terms (enrichment ≥ 5)	17	0

#### Scenario 2 – Multiple pairs of test/reference samples are available with no matching information

By ignoring matching information, we aggregated all test data (lung cancer) into one pool and all reference data (lung normal) into another pool, applied informME on the pooled data, and selected 450 genes as before using our JSD-based Fisher scheme (Additional file 2: Table S2). We also applied DSS-general on the data pairs and selected 450 genes based on the number of identified differentially methylated CpG sites within their promoters (Additional file 2: Table S3). The GO annotation results summarized in Table 1 (for details, see Additional file 2, Table S5b) were similar to the ones obtained in Scenario 1. Our method produced 167 GO terms, with 29 of them having enrichment of at least 5, whereas DSS produced only 3 GO terms with only 1 having enrichment of at least 5.

#### Scenario 3 – Only one pair of test/reference samples is available

To investigate this scenario, we separately applied informME on each matched pair of our WGBS data. By following our gene ranking scheme, we ranked genes using the average JSD score over all GUs that overlap a gene’s promoter, since we do not have replicate reference data in this case (Additional file 2: Table S6). This provided a target list of 450 genes for GO analysis. We also applied DSS-single on each matched pair and selected 450 genes as before based on the number of identified differentially methylated CpG sites within their promoters (Additional file 2: Table S6). For each normal/cancer pair, GO enrichment analysis produced the results summarized in Table 1 (for details, see Additional file 2, Table S7), which were again similar to the results obtained in the previous two scenarios. In the case of the (lungcancer-1, lungnormal-1) pair, our approach produced 176 GO terms, with 31 of them having enrichment of at least 5, whereas DSS produced 68 GO terms with only 9 having enrichment of at least 5. Moreover, in the case of the (lungcancer-2, lungnormal-2) pair, informME produced 148 GO terms, whereas DSS produced only 2 GO terms with none of these terms having enrichment of at least 5, compared to 25 such GO terms identified by informME. Finally, in the case of the (lungcancer-3, lungnormal-3) pair, informME produced 159 GO terms, whereas DSS produced 42 GO terms with none of these terms having enrichment of at least 5, compared to 17 such GO terms identified by informME.

### Methylation data analysis

We now illustrate the effectiveness of informME in procuring information about the methylation status of the epigenome within different genomic features and at multiple scales. We do so by analyzing our matched lung normal/cancer WGBS samples.

For each sample group (normal or cancer), we computed the distributions of aggregate GU classifications over the entire genome in terms of methylation level and entropy, as well as within enhancers, promoters, gene bodies, CGIs, and CGI shores (Additional file 1: Figures S6 and S7). We also computed the distributions of aggregate differential GU classifications among all cancer/normal comparisons in terms of methylation level and entropy (Additional file 1: Figures S8 and S9). We obtained a list of enhancers from the VISTA enhancer browser [50] by using all human (hg19) positive enhancers that show reproducible expression in at least three independent transgenic embryos. We defined promoter regions as sequences flanking 2-kb on either side of TSSs, which we determined by using the R Bioconductor package TxDb.Hsapiens.UCSC.hg19.knownGene. Finally, we downloaded a list of gene bodies from the UCSC genome browser (https://genome.ucsc.edu) and a list of CGIs from [51], whereas we defined CGI shores as sequences flanking 2-kb on either side of CGIs.

The distributions of aggregate GU classifications in terms of methylation level and entropy (Additional file 1: Figures S6 and S7) are in agreement with the known fact that the genome is mostly methylated in normal cells, except within CGIs, which are more likely to be unmethylated than methylated, as well as with the fact that cancer cells exhibit global hypomethylation. Moreover, these distributions show that, in addition to global hypomethylation, cancer cells can locally exhibit hypermethylation within certain genomic features. However, the distributions also demonstrate that a significant percentage of GUs within enhancers, promoters, gene bodies, and CGI shores (and to a lesser extend within CGIs) exhibit variable (mixed, highly mixed, or bistable) methylation, which noticeably increases in cancer.

The distributions of aggregate GU differential classifications (Additional file 1: Figures S8 and S9) demonstrate that the methylation state within most GUs in normal cells is weakly ordered/disorded. However, a significant percentage of GUs are ordered or disordered within promoters, are disordered within enhancers, and ordered within CGIs. Moreover, these distributions show appreciable global shift towards disordered states in cancer. However, a closer look of the results reveals that, although a large percentage (more than 40%) of GUs within enhancers, promoters, gene bodies, CGIs, and CGI shores are hyperentropic in cancer, a significant percentage (between 16% and 20%) becomes hypoentropic as well.

**Fig. 7:**
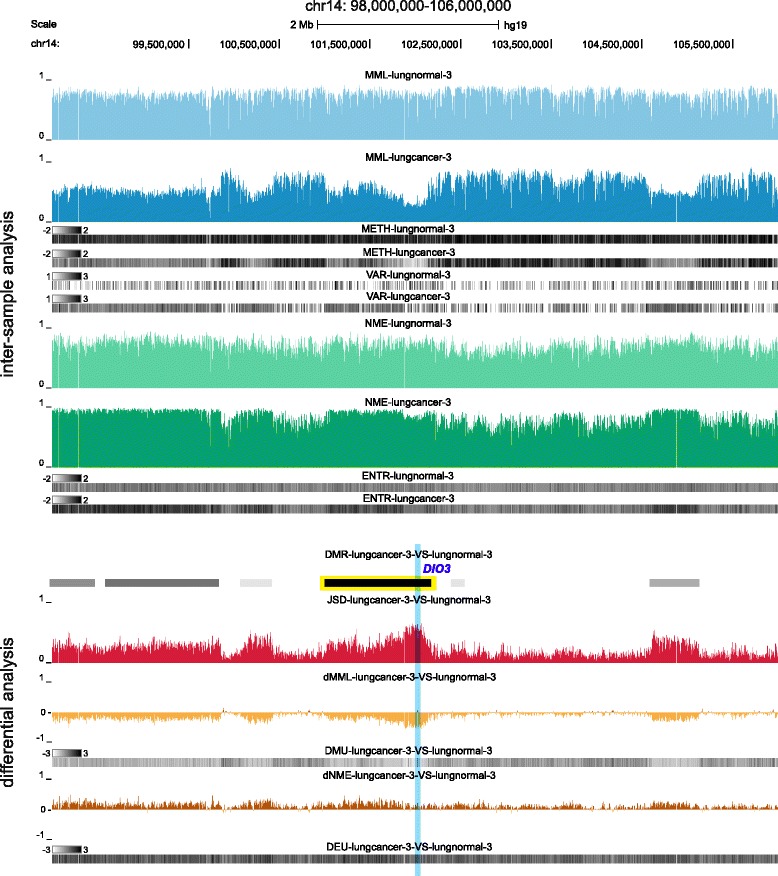
UCSC genome browser example of large-scale inter-sample and differential analysis of the matched WGBS sample pair (lungcancer-3, lungnormal-3) using informME. See Additional file 1, Section 9, for information about the depicted tracks. The highlighted DMR contains *DIO3*, a developmentally critical gene implicated in lung cancer and placed at the top of the list of ranked genes produced by informME

**Fig. 8:**
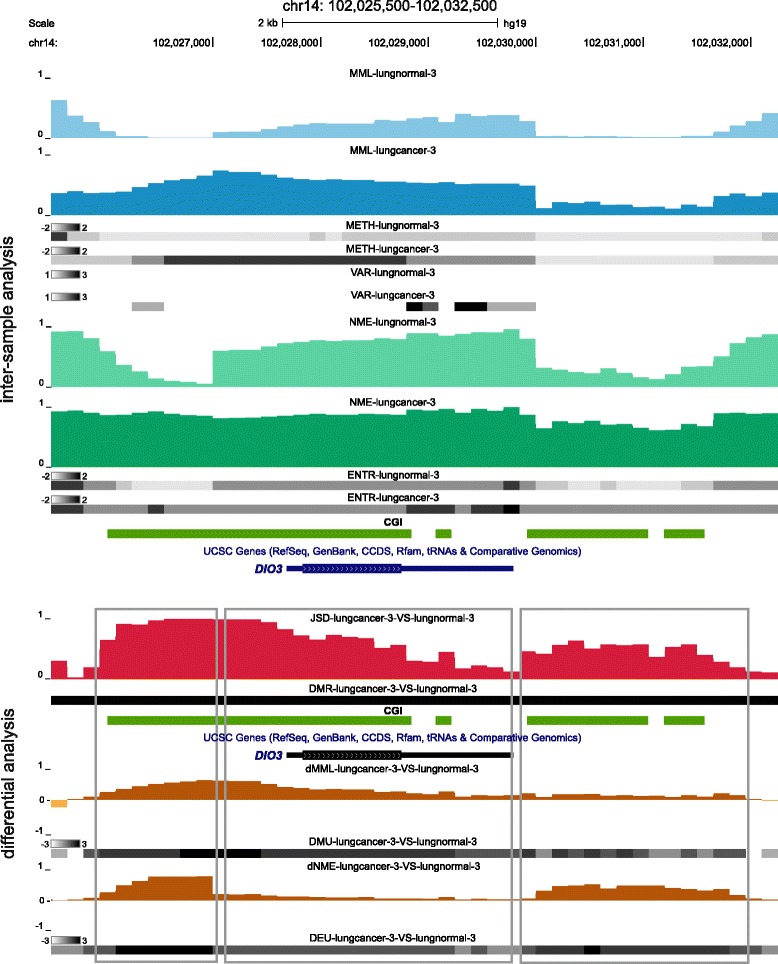
Local-scale version of the UCSC genome browser example depicted in Fig. 7 showing the methylation status within the genomic location of *DIO3*. See Additional file 1, Section 9, for information about the depicted tracks. Note that differential methylation activity in real data can be primarily driven by differences in mean methylation level (see II), entropy (see III), or both (see I)

informME can produce high resolution inter-sample and differential information about methylation within a genomic region. To illustrate this, we depict in Figs. 7 and 8 results for our matched (lungcancer-3, lungnormal-3) pair generated by informME within two genomic regions at two different scales: a large scale (8-Mb) genomic region within chr14 (98,000,000-106,000,000), depicted in Fig. 7, and a much smaller (7-kb) local genomic region within chr14 (102,025,500-102,032,500), depicted in Fig. 8. Most GUs within the genomic region depicted in Fig. 7 in the lungnormal-3 sample are partially or highly methylated with only a small number being partially or highly unmethylated (MML and METH tracks). However, a few GUs are sparsely classified as mixed, with a smaller number classified as highly mixed or bistable (VAR track). In addition, most GUs are moderately or highly disordered with some GUs being moderately or highly ordered (NME and ENTR tracks). Notably, lungcancer-3 exhibits global loss in mean methylation level (MML, dMML, and DMU tracks), a noticeable increase in GUs classified as mixed, highly mixed, or bistable (VAR tracks), and a gain in entropy (NME, ENTR, dNME, and DEU tracks). These differences drive high Jensen-Shannon distance values within a large number of GUs (JSD track), which lead to many differentially methylated regions (DMR track). The DMR highlighted by yellow in Fig. 7 contains *DIO3*, a critical developmental gene whose genomic location is highlighted by blue. This gene has been ranked 1-st in the list of ranked genes produced by informME (Additional file 2: Table S2, third list) and its genomic locus has been recently implicated in lung cancer [52, 53].

A closer inspection of the local region highlighted by blue in Fig. 7 reveals that the lung cancer sample exhibits gain in mean methylation level (MML, dMML, and DMU tracks), as well as in entropy (NME, ENTR, dNME, and DEU tracks), which result in significant Jensen-Shannon distance values (JSD track); see Fig. 8. Moreover, the results indicate that the CGIs within the genomic locus of *DIO3* are hypermethylated in lung cancer. This is in direct contrast to the hypomethylation observed at a larger scale, but in agreement with recent findings regarding the methylation state of *DIO3* in lung cancer [53]. With respect to methylation stochasticity, Fig. 8 shows an entropy gain in lung cancer, although this gain is significant only within the first 1/3 of the first CGI (see I), as well as within the third and the fourth CGIs (see III). Finally, Fig. 8 illustrates our previous point that differential methylation activity in real data can be primarily driven by differences in mean methylation level (see II), entropy (see III), or both (see I).

### Importance of JSD for differential methylation analysis

To demonstrate the importance of modeling methylation stochasticity in real data using joint probability distributions and identifying differential activity by employing the JSD, we investigated the possibility of finding genes with large average JSD values but small average absolute dMML values within their promoters in our lung data. We did so by first ranking all genes in two separate lists, with the genes in the first list ranked in terms of decreasing average absolute dMML values within their promoter regions and the genes in the second list ranked in terms of decreasing average JSD values. We then scored a gene using the ratio of its ranking in the mean-based list to its ranking in the JSD-based list, and used these scores to produce a new ranked list with higher ranked genes being characterized by larger average JSD values but smaller average absolute dMML values within their promoter regions (Additional file 2: Table S8).

**Fig. 9:**
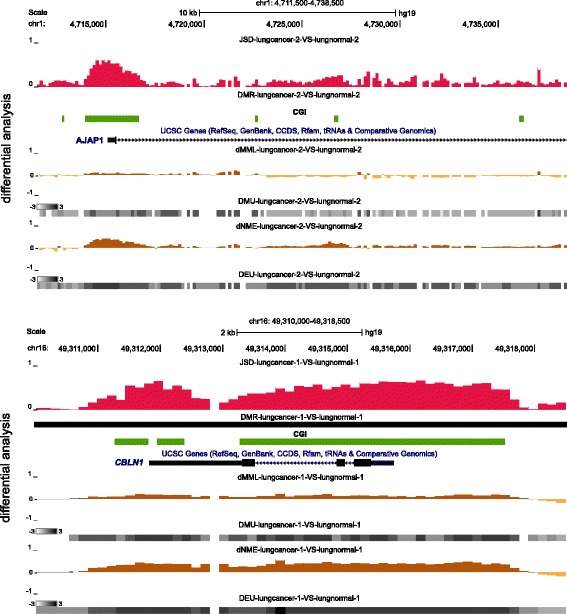
UCSC genome browser examples of *AJAP1* and *CBLN1*, two genes implicated in lung cancer with promoters exhibiting low levels of differential mean methylation between lung normal and lung cancer but large Jensen-Shannon distances. See Additional file 1, Section 9, for information about the depicted tracks

We identified many genes with this property that have been implicated in lung cancer, such as *AJAP1*, *CBLN1*, *FOXC2*, *OLIG2*, *POU3F3*, *SALL3*, and *SOX1*. For example, the genomic regions depicted in Fig. 9 contain *AJAP1* and *CBLN1*, which are respectively ranked 16-th and 14-th in the JSD-based lists of ranked genes obtained by informME in the case of the lungcancer-2-VS-lungnormal-2 and lungcancer-1-VS-lungnormal-1 comparisons (Additional file 2: Table S8). These regions are characterized by appreciable JSD values (JSD tracks) associated with very low differences in MML (dMML tracks) and moderate differences in NME (dNME tracks). Notably *AJAP1* is ranked 2262-nd in the corresponding ranked list of genes obtained by DSS, whereas *CBLN1* is ranked 1054-th (Additional file 2: Tables S6a and S6b, second lists). Note that the first region is not inside a DMR, which demonstrates the fact that DMR detection can miss important differential activity in methylation that appears at smaller scales.

Our previous results corroborate our claim that inter-sample and differential analysis of methylation stochasticity requires calculation of joint PMFs of methylation activity within regions of the genome and should not be based on marginal analysis, since such an analysis may be blind to important statistical behavior of methylation. In particular, differential analysis must be performed by comparing entire probability distributions and not just means, since two PMFs located at the same mean may have different shapes, indicating a differential behavior that is due to high-order statistical factors (see also Fig. 3).

### Implementation

We have implemented the previous methods for methylation analysis in informME, a publicly available software package written in MATLAB, C++ and R. The package is available under a GPL-3.0 license and can be downloaded from GitHub (https://github.com/GarrettJenkinson/informME).

informME produces results stored in bedGraph genomic tracks (Additional file 1: Section 9) that can be visualized using a genome browser, such as the UCSC genome browser (https://genome.ucsc.edu). For a given species (e.g., human, mouse, etc), a reference genome is first analyzed using MATLAB to compute, among other things, the location of CpG sites, the CpG density of each CpG site, and the distance between neighboring CpG sites. BAM files of WGBS reads aligned to the reference genome are then passed to a matrix generation algorithm of MATLAB, which performs methylation calling and places the data in convenient matrix data structures that enable rapid statistical estimation of the Ising model parameters. This information is then passed to the next step, which estimates the parameters of the 1D Ising model, given by (1)–(4), within each 3-kb estimation region ${\mathcal {R}}_{k}$ of the genome via maximum-likelihood. For computational efficiency, the iterative algorithms that calculate the partition functions and marginalized joint probability distributions required in this step have been written in C++ using the MATLAB executable (MEX) API. Computation of the partition function requires use of large numbers and, for this reason, standard double-precision arithmetic is not sufficient. Thus, informME employs arbitrary precision arithmetic to ensure numerical accuracy. In the C++ code, arbitrary precision computations are facilitated by the MPFR C library for multi-precision floating-point computations with correct rounding (http://www.mpfr.org), along with the EIGEN C++ template library for linear algebra (http://eigen.tuxfamily.org).

Subsequently, informME performs methylation analysis of a single WGBS sample by computing a number of statistical summaries of the methylation state, including MMLs and NMEs, as well as mean and entropy based classifications. Moreover, informME can perform differential methylation analysis between a test and a reference sample by computing a number of statistical summaries of the differential methylation state, including differences in MMLs and NMEs, JSDs, and differential mean level and entropy based classifications. Finally, informME is currently equipped with two post-processing R functions: jsDMR, a utility that performs JSD-based DMR detection, and jsGrank, a utility that uses the JSD to rank all genes in the human genome in terms of their epigenetic discordance between test and reference WGBS samples.

We evaluated the time and memory requirements of informME versus that of DSS using our (lungcancer-3, lungnormal-3) pair of samples. The results, which we summarize in Additional file 1: Table S1, show that informME is overall computationally more expensive than DSS, requiring about 6.5 times the CPU time of DSS but less than 1/4 of the maximum RAM required by DSS. Note, however, that the additional cost in CPU time results in several important benefits: joint PMFs are computed within GUs, which allows computation of any statistical summary of interest beyond the mean, statistically valid results are produced in the presence of correlations (which are always present in methylation data), and additional information-theoretic quantities are calculated that can be effectively used in inter-sample and differential methylation analysis. We should finally point out that the highly parallelizable structure of informME means that access to a computer cluster can reduce implementation time below that of DSS. Consequently, in our extensive experimentation on a computing cluster, we found that the time a user must spend waiting for informME to process a WGBS experiment (∼ 1 day) is far less than the time it takes to sequence and demux the samples (and much less time than wet lab experiments take to produce the samples). We thus contend that waiting on accurate and comprehensive bioinformatics modeling of methylation data is completely justified and reasonable in the context of large, expensive, and inherently time-consuming genome-wide sequencing studies.

## Discussion

The Ising model was originally introduced in statistical physics as a model of ferromagnetism [25]. Despite its wide-spread use in many fields of science and engineering as a model that accounts for statistical correlations, it has only been recently adopted for modeling correlations in DNA methylation data [22]. The MATLAB, C++, R-package we have developed and discussed in this paper within the framework of informME includes methods for fitting the Ising model to WGBS data and for extracting information from such data in inter-sample or differential analysis methylation studies.

Previous simulation studies have offered strong evidence that the Ising model can perform exceptionally well in accurately estimating measures of methylation stochasticity, such as mean methylation levels and normalized methylation entropies, even at low coverage [22]. This is in sharp contrast to existing empirical approaches to methylation analysis, which do not perform well with highly stochastic methylation data and at low coverage.

Building upon this foundation, the results presented in this paper, using human lung normal/cancer methylation data, clearly demonstrate the potential of informME as a powerful statistical methylation analysis tool. We attribute this result to the fact that informME performs methylation analysis by effectively taking into account the massive amount of statistical information available in WGBS data, which is largely ignored by existing methods for methylation analysis based on marginal or mean analysis, such as DSS. In addition, informME models methylation within GUs using joint probability distributions that encapsulate high-order statistical factors, for example NME and JSD, which cannot be captured by a marginal statistical approach. This type of marginal analysis was shown here not to be sufficient for fully characterizing methylation stochasticity, consistent with recent findings [20, 22].

The Ising model was justified by a maximum entropy approach by assuming that the means and nearest-neighbor correlations are all that can be reliably observed genomewide by current WGBS technology. However, third generation sequencing promises longer reads, which may reveal the importance of taking into account higher-order statistical information. By following a similar maximum entropy approach, the methodology discussed in this paper can be extended to the more general class of Gibbs distributions that include additional terms in their energy functions. However, this approach will introduce more parameters in the model to be estimated from available data, which will in turn increase the statistical complexity of the problem and require availability of higher coverage data. Finally, the promise of long reads from third generation sequencing holds great potential for providing fully observed data within a genomic region. This will lead to a convex maximum-likelihood estimation problem that can be rapidly solved by an efficient convex optimization algorithm.

## Conclusion

In this paper, we presented informME, a novel information-theoretic pipeline for inter-sample and differential methylation analysis of WGBS data. In contrast to most existing methods for methylation analysis, informME considers all information available in methylation reads, takes into account statistical dependencies between the methylation states of CpG sites, and quantifies methylation stochasticity not by simple means and variances at individual CpG sites but by using joint probability distributions over the methylation states.

Here we showed that the probability mass function of methylation within a region of the genome can be approximated by the 1D Ising model of statistical physics and presented algorithms for computing the associated partition function and for calculating marginal probabilities, which are critical to the maximum likelihood estimation problem central to informME. In addition, we confirmed the identifiability of the underlying parameters and provided details of the methods used by informME to calculate the probability mass function of the methylation level within a genomic unit. We also developed inter-sample and differential classification schemes for the methylation level and the Shannon entropy within genomic units, and presented a new method for detecting DMRs using the Jensen-Shannon distance between two probability distributions. Moreover, we discussed a method that uses this distance to rank genes based on observed epigenetic discordance within their promoters. We also evaluated the appropriateness of the particular Ising model used by informME by employing Akaike’s information criterion. We finally demonstrated the clear superiority of informME over DSS and metilene, two recently proposed methods for differential analysis of bisulfite sequencing data, and illustrated its effectiveness in producing information about the methylation state of the epigenome within different genomic features and at multiple scales. With the rapidly decreasing cost of sequencing and corresponding increases in the availability of WGBS technology, there will be ample opportunities to apply informME on a wide range of genomewide inter-sample and differential methylation studies. In the future, it will be important to explore further improvements to the Ising model and our information-theoretic framework, such as incorporating genomic SNP information into the formulation to aid in methylation quantitative trait loci (mQTL) analysis.

## Additional files

Additional file 1 Supplementary material. This file contains additional method descriptions and supplementary figures. (PDF 3,808 KB)

Additional file 2 Supplementary tables. This file contains supplementary tables summarizing our experimental results. (XLSX 10,320 KB)

## Abbreviations

AIC: Akaike’s information criterion
bp: Base pair
CGI: CpG island
dMML: Differential mean methylation level
DMR: Differentially methylated region
dNME: Differential normalized methylation entropy
FDR: False discovery rate
GO: Gene ontology
GU: Genomic unit
informME: information-theoretic analysis of methylation
JSD: Jensen-Shannon distance
KS: Kolmogorov-Smirnov
MML: Mean methylation level
MWU: Mann-Whitney U
NME: Normalized methylation entropy
PMF: Probability mass function
sJSD: Smoothed Jensen-Shannon distance
SQS: Statistical quality score
TSS: Transcription start site
WGBS: Whole genome bisulfite sequencing
