# Supplementary material for: An information-theoretic approach to the modeling and analysis of whole-genome bisulfite sequencing data
Source: BMC Bioinformatics. 2018 Mar 7;19:87. doi: 10.1186/s12859-018-2086-5 (PMC5842653; doi:10.1186/s12859-018-2086-5)
Supplement: Supplementary file 1 — Supplementary material. This file contains additional method descriptions and supplementary figures. (PDF 3,808 KB) [file 12859_2018_2086_MOESM1_ESM.pdf]

SUPPLEMENTARY MATERIAL

An information-theoretic approach to the modeling and analysis of whole-genome bisulfite sequencing data

G. Jenkinson, J. Abante, A. P. Feinberg, and J. Goutsias

1 PMF of methylation within a genomic region

We can approximately express the PMF of the methylation state within a genomic region $\mathcal{R}_k$ in terms of a 1D Ising model. To see why this is true, let us assume that $\mathcal{R}_k$ comprises R CpG sites $1, 2, \ldots, R$, which are labeled according to their order of appearance along the region. Note that the PMF of methylation within $\mathcal{R}_k$ satisfies

$$\begin{aligned} P_X(x_1, x_2, \ldots, x_R) &= P_X(x_2, x_3, \ldots, x_{R-1} \mid x_1, x_R) P_X(x_1, x_R) \\ &\simeq P_X(x_2, x_3, \ldots, x_{R-1} \mid x_1, x_R) P_X(x_1) P_X(x_R), \end{aligned} \tag{S1}$$

where we assume that X_1 and X_R are approximately statistically independent. This is a reasonable assumption considering the fact that, in most cases, the two CpG sites near the boundary of $\mathcal{R}_k$ are separated by many intermediate CpG sites. We can now show from Eqs. (1)–(4) of the Main Paper that

$$\begin{aligned} &P_X(x_2, x_3, \ldots, x_{R-1} \mid x_1, x_R) \\ &\quad \propto \exp\Big\{ \sum_{r=2}^{R-1} (\alpha_k + \beta_k \rho_r)(2x_r - 1) + \sum_{r=2}^{R} \frac{\gamma_k}{d_r}(2x_r - 1)(2x_{r-1} - 1) \Big\}. \end{aligned} \tag{S2}$$

Therefore, if we set

$$\alpha'_k = \frac{1}{2} \ln \frac{\Pr[X_1 = 1]}{1 - \Pr[X_1 = 1]} \quad \text{and} \quad \alpha''_k = \frac{1}{2} \ln \frac{\Pr[X_R = 1]}{1 - \Pr[X_R = 1]}, \tag{S3}$$

and use (S1) and (S2), we approximately obtain

$$\begin{aligned} P_X(x_1, x_2, \ldots, x_R) = \frac{1}{Z} \exp\Big\{ &\alpha'_k(2x_1 - 1) + \sum_{r=2}^{R-1} (\alpha_k + \beta_k \rho_r)(2x_r - 1) + \alpha''_k(2x_R - 1) \\ &+ \sum_{r=2}^{R} \frac{\gamma_k}{d_r}(2x_r - 1)(2x_{r-1} - 1) \Big\}, \end{aligned} \tag{S4}$$

where

$$\begin{aligned} Z = \sum_{\boldsymbol{u}} \exp\Big\{ &\alpha'_k(2u_1 - 1) + \sum_{r=2}^{R-1} (\alpha_k + \beta_k \rho_r)(2u_r - 1) + \alpha''_k(2u_R - 1) \\ &+ \sum_{r=2}^{R} \frac{\gamma_k}{d_r}(2u_r - 1)(2u_{r-1} - 1) \Big\}. \end{aligned} \tag{S5}$$

Notably, this is an Ising model, albeit with a smaller number of parameters when $R \geq 4$ (5 vs. $2R-1$) than the one without using Eqs. (3) and (4) of the Main Paper. Note also that parameters α'_k and α''_k account for boundary effects that occur when restricting the Ising model associated with the entire genome to the Ising model within $\mathcal{R}_k$.

2 Computing the partition function

In general, evaluating the PMF $P_X(x_1, x_2, \ldots, x_R)$ within a genomic region $\mathcal{R}_k$ is not straightforward. This is due to the fact that the partition function Z cannot be easily computed since it is a sum over a large number (2^R) of distinct states, even for a moderate number of CpG sites R. However, since the PMF is given by (S4) and (S5), we can show that

$$P_X(x_1, x_2, \ldots, x_R) = \frac{1}{Z} \prod_{r=1}^{R-1} \phi_r(x_r, x_{r+1}), \tag{S6}$$

where

$$Z = \sum_{u_1=0}^{1} \sum_{u_2=0}^{1} \cdots \sum_{u_R=0}^{1} \prod_{r=1}^{R-1} \phi_r(u_r, u_{r+1}), \tag{S7}$$

with

$$\begin{aligned}
&\phi_1(x_1, x_2) \\
&\qquad = \exp\left\{\alpha'_k(2x_1-1) + (\alpha_k + \beta_k \rho_2)(2x_2-1) + \frac{\gamma_k}{d_2}(2x_1-1)(2x_2-1)\right\}, \\
&\phi_r(x_r, x_{r+1}) \\
&\qquad = \exp\left\{(\alpha_k + \beta_k \rho_{r+1})(2x_{r+1}-1) + \frac{\gamma_k}{d_{r+1}}(2x_r-1)(2x_{r+1}-1)\right\}, \\
&\qquad\qquad\qquad\qquad\qquad\qquad\qquad \text{for } 2 \leq r \leq R-2, \\
&\phi_{R-1}(x_{R-1}, x_R) = \exp\left\{\alpha''_k(2x_R-1) + \frac{\gamma_k}{d_R}(2x_{R-1}-1)(2x_R-1)\right\}.
\end{aligned} \tag{S8}$$

Equations (S7) and (S8) can be employed to compute the partition function using the following iteration:

$$\begin{aligned}
Z_R(x) &= 1, \quad \text{for } x = 0, 1 \\
Z_r(x) &= \phi_r(x, 0) Z_{r+1}(0) + \phi_r(x, 1) Z_{r+1}(1), \\
&\qquad\qquad \text{for } x = 0, 1, \; r = R-1, R-2, \ldots, 1 \\
Z &= Z_1(0) + Z_1(1).
\end{aligned} \tag{S9}$$

This allows evaluation of the probability $P_X(x_1, x_2, \ldots, x_R)$ of *any* methylation state within $\mathcal{R}_k$ using (S6)–(S8).

3 Computing marginal PMFs

Fitting the Ising methylation model to available WGBS data requires evaluation of marginal PMFs within a genomic region $\mathcal{R}_k$ of the form $P_X(x_{q:q+s}) = P_X(x_q, x_{q+1}, \ldots, x_{q+s})$, where q and s are such that $1 \leq q \leq q+s \leq R$. From (S6) and (S9), we can show that

$$P_X(x_1, x_2, \ldots, x_R) = z_1(x_1) \prod_{r=1}^{R-1} z_{r+1}(x_{r+1} \mid x_r), \tag{S10}$$

where

$$z_1(x_1) = \frac{Z_1(x_1)}{Z}, \tag{S11}$$

and

$$z_{r+1}(x_{r+1} \mid x_r) = \frac{\phi_r(x_r, x_{r+1}) Z_{r+1}(x_{r+1})}{Z_r(x_r)}, \quad \text{for } r = 1, 2, \ldots, R-1. \tag{S12}$$

This result provides an alternative representation of the 1D Ising model in terms of an *inhomogeneous* Markov chain with initial probability $z_1(x_1)$ and transition probabilities $z_{r+1}(x_{r+1} \mid x_r)$. From (S10), we have that

$$\begin{aligned} P_X(x_{1:q+s}) &= z_1(x_1) \prod_{r=1}^{q+s-1} z_{r+1}(x_{r+1} \mid x_r) \\ &= z_1(x_1) \prod_{r=1}^{q-1} z_{r+1}(x_{r+1} \mid x_r) \prod_{r=q}^{q+s-1} z_{r+1}(x_{r+1} \mid x_r), \end{aligned} \tag{S13}$$

which implies

$$P_X(x_{q:q+s}) = w_q(x_q) \prod_{r=q}^{q+s-1} z_{r+1}(x_{r+1} \mid x_r), \tag{S14}$$

where

$$w_q(x_q) = \sum_{x_1} \sum_{x_2} \cdots \sum_{x_{q-1}} z_1(x_1) \prod_{r=1}^{q-1} z_{r+1}(x_{r+1} \mid x_r). \tag{S15}$$

Moreover, from (S11), (S12), and (S15), note that

$$\begin{aligned} w_q(x_q) &= \frac{Z_q(x_q)}{Z} \sum_{x_1} \sum_{x_2} \cdots \sum_{x_{q-1}} \prod_{r=1}^{q-1} \phi_r(x_r, x_{r+1}) \\ &= \frac{Z_q(x_q)}{Z} \sum_{x_{q-1}} \cdots \left[\sum_{x_2} \left[\sum_{x_1} \phi_1(x_1, x_2) \right] \phi_2(x_2, x_3) \right] \cdots \phi_{q-1}(x_{q-1}, x_q). \end{aligned} \tag{S16}$$

This implies that

$$w_q(x_q) = \frac{1}{Z} Z_q(x_q) \widetilde{Z}_q(x_q), \tag{S17}$$

where $\widetilde{Z}(x_q)$ is computed by

$$
\begin{aligned}
\widetilde{Z}_1(x) &= 1, \quad \text{for } x = 0, 1 \\
\widetilde{Z}_r(x) &= \phi_{r-1}(0, x)\widetilde{Z}_{r-1}(0) + \phi_{r-1}(1, x)\widetilde{Z}_{r-1}(1), \\
&\qquad\qquad \text{for } x = 0, 1, \ r = 2, 3, \ldots, q.
\end{aligned}
\tag{S18}
$$

Finally, by combining (S11), (S12), (S14), and (S15), we obtain

$$
P_X(x_{q:q+s}) = \frac{1}{Z}\, Z_{q+s}(x_{q+s})\widetilde{Z}_q(x_q) \prod_{r=q}^{q+s-1} \phi_r(x_r, x_{r+1}),
\tag{S19}
$$

which provides a formula for calculating the marginal PMF $P_X(x_{q:q+s})$ using the iterations in (S9) and (S18). Note that computation of the marginal PMFs $P_X(x_{q:q+s})$ requires the iterations (S9) and (S18) to be performed only once as long as the intermediate values $Z_r(x), \widetilde{Z}_r(x), x = 0, 1, r = 1, \ldots, R$, are stored.

4 Identifiability

It is worth pointing out here that the PMF $P_X(\boldsymbol{x} \mid \boldsymbol{\theta}_k)$ within a genomic region $\mathcal{R}_k$ forms a five-parameter exponential family of distributions with natural sufficient statistic given by [see (S4) and (S5)]

$$
\boldsymbol{S}(\boldsymbol{X}) = \begin{pmatrix} S_1(\boldsymbol{X}) \\ S_2(\boldsymbol{X}) \\ S_3(\boldsymbol{X}) \\ S_4(\boldsymbol{X}) \\ S_5(\boldsymbol{X}) \end{pmatrix} = \begin{pmatrix} 2X_1 - 1 \\ \sum_{r=2}^{R-1} (2X_r - 1) \\ 2X_R - 1 \\ \sum_{r=2}^{R-1} \rho_r (2X_r - 1) \\ \sum_{r=2}^{R} \dfrac{(2X_r - 1)(2X_{r-1} - 1)}{d_r} \end{pmatrix}.
\tag{S20}
$$

Since the CpG density ρ_r depends in general on r (it changes from a CpG site to the next), $\{S_1(\boldsymbol{X}), S_2(\boldsymbol{X}), S_3(\boldsymbol{X}), S_4(\boldsymbol{X}), S_5(\boldsymbol{X}), 1\}$ are linearly independent with positive probability, due to the fact that the Ising model assigns positive probabilities over the methylation state-space. In this case, the exponential family has rank 5 and the parameter vector $\boldsymbol{\theta}_k$ is *identifiable* according to Theorem 1.6.4 in [1].

5 Computing the PMF of methylation level

For GUs containing at most 18 CpG sites, we calculate the PMF $P_L(\ell)$ of the methylation level L using the *exact* summation formula in Eq. (10) of the Main Paper. For GUs with more than 18

CpG sites, we estimate $P_L(\ell)$ by combining Monte Carlo sampling with the maximum entropy principle [5, 6]. To do so, we first draw M samples $\boldsymbol{x}^{(1)}, \boldsymbol{x}^{(2)}, \ldots, \boldsymbol{x}^{(M)}$ from the Ising distribution $P_X(\boldsymbol{x})$ associated with the GU, which we then use to produce M samples $\ell_1, \ell_2, \ldots, \ell_M$ of the methylation level L by

$$\ell_m = \frac{1}{K} \sum_{k=1}^{K} x_k^{(m)}, \quad \text{for } m = 1, 2, \ldots, M. \tag{S21}$$

We then estimate the first Q non-central moments $\mathrm{E}[L^q]$, $q = 1, 2, \ldots, Q$, of L by means of

$$\mu_q = \frac{1}{M} \sum_{m=1}^{M} \ell_m^q, \quad \text{for } q = 1, 2, \ldots, Q. \tag{S22}$$

Finally, we approximate $P_L(\ell)$ by a probability distribution over the values of L that maximizes the Shannon entropy $-\sum_\ell P_L(\ell) \log_2 P_L(\ell)$, subject to the moment constraints $\sum_\ell \ell^q P_L(\ell) = \mu_q$, for $q = 1, 2, \ldots, Q$.

Note that we can rapidly draw *exact* samples from the Ising probability distribution $P_X(\boldsymbol{x})$ within a GU with K CpG sites by noting that $P_X(\boldsymbol{x})$ is given by Eqs. (S10)–(S12), with $R = K$. Equation (S10) implies that the methylation sequence $\{X_1, X_2, \ldots, X_K\}$ is a first-order non-homogeneous Markov chain with initial and transition probabilities given by (S11) and (S12), respectively. As a consequence, we can obtain an exact realization $\boldsymbol{x}$ by iteratively drawing samples $x_1, x_2, \ldots, x_K$ from these conditional probability distributions.

In (S22), we use $M = 2^{17} = 131{,}072$ Monte Carlo samples and set $Q = 4$ (i.e., we use Monte Carlo sampling to estimate the first four non-central moments of L). We investigated a number of Q values ($Q = 3, 4, 5, 6$) and determined that $Q = 4$ leads to robust Monte Carlo estimation of the probability distribution $P_L(\ell)$ within GUs with at most 18 CpG sites for which we can exactly compute $P_L(\ell)$ using the summation in Eq. (10) of the Main Paper. We also determined the value of M by noting that exact summation requires evaluation of at most $2^{18} = 262{,}144$ probabilities $P_X(\boldsymbol{x})$ when $K \leq 18$, a computation that we found to be feasible on our computer system. However, when $K > 18$, this calculation becomes increasingly less efficient, and that is why we switch from exact summation to Monte Carlo estimation. On our computer system, each probability evaluation is done in half the time required for drawing a sample from $P_X(\boldsymbol{x})$. As a consequence, when $K \leq 18$, exact summation is at least as computationally efficient as drawing $2^{17} = 131{,}072$ Monte Carlo samples, and this observation guides us to set $M = 2^{17}$. Finally, note that there are 11,273,889 GUs with at least 1 CpG site and at most 18 CpG sites in the human genome, but only 30,024 GUs with the number of CpG sites ranging from 19 to 44 (Additional file 2: Table S1). As a consequence, we compute the PMF of the methylation level L by exact summation for the vast majority (99.73%) of the GUs with at least one CpG site.

6 Single-sample classification of GUs

6.1 Methylation-based classification

To effectively summarize the status of the methylation level L within a GU, we compute the following probabilities

$$\begin{aligned} p_1 &= \Pr[0 \leq L \leq 0.25] \\ p_2 &= \Pr[0.25 < L < 0.5] + \tfrac{1}{2}\Pr[L = 0.5] \\ p_3 &= \tfrac{1}{2}\Pr[L = 0.5] + \Pr[0.5 < L < 0.75] \\ p_4 &= \Pr[0.75 \leq L \leq 1] \end{aligned} \qquad \text{(S23)}$$

and employ these probabilities to classify the GU using the scheme depicted in Fig. S1. This scheme classifies a GU as being *unmethylated* if more than 60% of its copies in a cell population have methylation level below 50% (i.e., if $p_1 + p_2 > 0.6$). In particular, the GU is classified as being *partially unmethylated* if no more than 60% of these copies have methylation level below 25% (i.e., if $p_1 \leq 0.6$); otherwise, it is classified as being *highly unmethylated*. Similarly, the GU is classified as being *methylated* if more than 60% of its copies in a cell population have methylation level above 50% (i.e., if $p_1 + p_2 < 0.4$, which implies that $p_3 + p_4 > 0.6$). In particular, the GU is classified as being *partially methylated* if no more than 60% of these copies have methylation level above 75% (i.e., if $p_4 \leq 0.6$); otherwise, it is classified as being *highly methylated*.

If a GU is neither unmethylated or methylated (i.e., if $0.4 \leq p_1 + p_2 \leq 0.6$), then it is classified as being *mixed* if no more than 40% of its copies with methylation level below 50% have methylation level of at most 25% [i.e., if $p_1/(p_1 + p_2) \leq 0.4$] and no more than 40% of its copies with methylation level above 50% have methylation level of at least 75% [i.e., if $p_4/(p_3 + p_4) \leq 0.4$]. On the other hand, the GU is classified as being *highly mixed* if more than 40% but no more than 60% of its copies with methylation level below 50% have methylation level of at most 25% [i.e., if $0.4 < p_1/(p_1 + p_2) < 0.6$] and more than 40% but no more than 60% of its copies with methylation level above 50% have methylation level of at least 75% [i.e., if $0.4 < p_4/(p_3 + p_4) \leq 0.6$]. Finally, the GU is classified as being *bistable* if at least 60% of its copies with methylation level below 50% have methylation level of at most 25% [i.e., if $p_1/(p_1 + p_2) \geq 0.6$] and at least 60% of its copies with methylation level above 50% have methylation level above 75% [i.e., if $p_4/(p_3 + p_4) \geq 0.6$].

As we discuss in the Main Paper, the mixed and highly mixed GUs are characterized by appreciable methylation variability, whereas, bistable GUs are characterized by the highest possible variance in methylation level. For this reason, we refer to a GU that is mixed, highly mixed, or bistable as being *variably methylated*.

To see why a bistable GU is characterized by the highest possible variance, let us consider for example a highly mixed GU with N CpG sites in which the distribution of methylation level is uniform, and a bistable GU with probability distribution whose mass is equally distributed at methylation levels 0 and $1/N$. In both cases, the mean is given by $1/2$. However, the highly mixed GU is characterized by a variance of $(N + 2)/12N$, whereas the bistable GU is characterized by a variance of $1/4 \geq (N + 2)/12N$.

6.2 Entropy-based classification

To effectively summarize the status of the NME within a GU, we employ the following classification scheme:

$$\begin{array}{ll} \text{highly ordered} & 0 \leq h \leq 0.28 \\ \text{moderately ordered} & 0.28 < h \leq 0.44 \\ \text{weakly ordered/disordered} & 0.44 < h < 0.92 \\ \text{moderately disordered} & 0.92 \leq h < 0.99 \\ \text{highly disordered} & 0.99 \leq h \leq 1 \end{array} \tag{S24}$$

We determined the previous thresholds by studying the NME within a region containing one CpG site. In this case, the methylation level L assumes two possible values, 1 or 0, with probabilities p and $1 - p$, respectively. By focusing on the odds ratio $r = p/(1 - p)$, we consider the random variable L to be "moderately ordered" if $r \geq 10$ or $r \leq 1/10$, whereas we consider it to be "highly ordered" if $r \geq 20$ or $r \leq 1/20$. In the first case, $p \geq 0.9091$ or $r \leq 0.0909$, which corresponds to a maximum NME value of 0.44, whereas, in the second case, $p \geq 0.9524$ or $p \leq 0.0476$, which corresponds to a maximum NME value of 0.28. Furthermore, we consider L to be "moderately disordered" if $1/2 \leq r \leq 2$, whereas we consider it to be "highly disordered" if $1/1.2 \leq r \leq 1.2$. In the first case, $0.3333 \leq p \leq 0.6667$, which corresponds to a minimum NME value of 0.92, whereas, in the second case, $0.4545 \leq p \leq 0.5455$, which corresponds to a minimum NME value of 0.99.

7 Differential classification of GUs

7.1 Methylation-based differential classification

Using the PMF of the difference $D_L = L_t - L_r$ in methylation level within a GU between a test and a reference sample, we calculate the following probabilities:

$$\begin{aligned} q_1 &= \Pr[-1 \leq D_L \leq -0.55] \\ q_2 &= \Pr[-0.55 < D_L \leq -0.1] \\ q_3 &= \Pr[-0.1 < D_L < 0.1] \\ q_4 &= \Pr[0.1 \leq D_L < 0.55] \\ q_5 &= \Pr[0.55 \leq D_L \leq 1] \end{aligned} \tag{S25}$$

and use them to classify the GU by employing the scheme depicted in Fig. S2. It turns out that a GU is classified as being *isomethylated* if the absolute difference in methylation levels between two copies of the GU, one randomly chosen from the test sample and the other from the reference sample, is less than 0.1 more than 55% of the time (i.e., if $q_3 > 0.55$). When the GU is not isomethylated, it is classified as being *hypomethylated* if the difference in methylation levels between its two randomly chosen copies is no more than -0.1 more than 55% of the time (i.e., if $\Pr[D_L \leq -0.1 \mid |D_L| \geq 0.1] > 0.55$, which implies that $(q_1 + q_2)/(1 - q_3) > 0.55$). In particular, the GU is classified as being *strongly hypomethylated* if the difference in methylation levels is less than -0.55 more than 55% of the time (i.e., if $q_1 > 0.55$). On the other hand, it is

classified as being *moderately hypomethylated* if the difference in methylation levels is between -1 and -0.1 more than 55% of the time with the difference being smaller than -0.55 no more than 55% of the time (i.e., if $q_1 \leq 0.55$ but $q_1 + q_2 > 0.55$). Finally, the GU is classified as being *weakly hypomethylated* if the difference in methylation levels is between -1 and -0.1 no more than $(1 - q_3) \times 55\%$ but no more than 55% of the time (i.e., if $0.55 \times (1 - q_3) < q_1 + q_2 \leq 0.55$). Similar remarks apply for classifying the GU as being hypermethylated.

7.2 Entropy-based differential classification

By computing the difference $D_h = h_t - h_r$ in NMEs between a test and a reference sample within a GU, we classify the GU into one of seven classes using the following simple thresholding scheme:

$$\begin{array}{ll} \text{strongly hypoentropic} & -1 \leq D_h \leq -0.5 \\ \text{moderately hypoentropic} & -0.5 < D_h \leq -0.3 \\ \text{weakly hypoentropic} & -0.3 < D_h \leq -0.05 \\ \text{isoentropic} & -0.05 < D_h < 0.05 \\ \text{weakly hyperentropic} & 0.05 \leq D_h < 0.3 \\ \text{moderately hyperentropic} & 0.3 \leq D_h < 0.5 \\ \text{strongly hyperentropic} & 0.5 \leq D_h \leq 1 \end{array} \tag{S26}$$

We choose the previous thresholds to approximately be in agreement with the thresholds 0.28 and 0.44 used for entropy-based classification of GUs in single samples; see (S24). Indeed, we consider a test sample to be isoentropic to a reference sample within a GU if $0 \leq |h_t - h_r| < 0.05$, to be weakly hyperentropic if $0.05 \leq h_t - h_r < 0.28 + 0.05 \simeq 0.3$, to be moderately hyperentropic if $0.3 \leq h_t - h_r < 0.44 + 0.05 \simeq 0.5$, strongly hyperentropic if $0.5 \leq h_t - h_r \leq 1$, and similarly for being weakly/moderately/strongly hypoentropic.

8 A critique of metilene

8.1 Statistics in metilene

Metiline, a recently proposed method for differential methylation analysis [4], performs hypothesis testing using the Mann-Whitney U (MWU) test (also known as the Wilcoxon ranksum test) or a two-dimensional extension of the Kolmogorov-Smirnov (KS) test [3]. Similarly to most methods of methylation analysis published in the literature, such as DSS, metilene fails to consider the joint statistical properties of DNA methylation and does not test against a null hypothesis that accurately reflects the goal of differential analysis. Moreover, and inconsistently with other methods (such as DSS), metilene does not account for the data generation process and variations in depth of coverage that are always present in sequencing data.

Most "marginal" methods of methylation analysis of sequencing data require two random variables to be observed at each CpG site n of the genome: the number M_n of methylated observations as well as the total number of observations (coverage) C_n. In this case, the data generation process at each CpG site can be modeled as a binomial distribution and the marginal probability of the CpG site to be methylated can be estimated by using the maximum likelihood estimator $\widehat{p}_n = M_n/C_n$. Clearly, the quality of the resulting estimate increases as the coverage C_n

increases (i.e., the width of the confidence interval for this estimate shrinks as the number of observations increases). For small C_n, the maximum likelihood estimator $\widehat{p}_n$ is highly unreliable. However, it asymptotically converges to the true marginal probability of methylation as the coverage increases. It is therefore critical to take the coverage C_n into account when formulating a statistical procedure to detect methylation differences. Surprisingly, metilene uses only the empirical estimator $\widehat{p}_n$ and thus it cannot account for the data generation process and the resulting uncertainty in the estimate. For example, a value $\widehat{p}_n = 1$ that comes from a single observation and results in a 95% confidence interval of $[0.025, 1]$ (using the binomial distribution) would be treated by metilene exactly the same as a value $\widehat{p}_n = 1$ that comes from 50 observations that results in a 95% confidence interval of $[0.93, 1]$. As a result, the statistical procedures employed by metilene are highly questionable regardless of its mode of operation or choice of the statistical hypothesis test used. We discuss this important issue in the following.

8.2 Differential analysis using the MWU test

When examining one CpG site at a time, say the n-th CpG site, the input to metilene consists of observations that are split into a test and a reference group. Specifically, the method uses the maximum likelihood estimators $\widehat{p}_n^{(t)}(k)$ and $\widehat{p}_n^{(r)}(l)$ to estimate the marginal probabilities of the n-th CpG site to be methylated in the k-th test and l-th reference samples, respectively. It then attempts to detect differential methylation at this CpG site by comparing the probability distributions $\widehat{p}_n^{(t)}$ and $\widehat{p}_n^{(r)}$ using a two-sample MWU test on the observed values. In the case of a predefined region of interest or a potential DMR, metilene performs MWU analysis by pooling the $\widehat{p}$ values associated with all observed CpG sites within the region of interest or DMR in each group (test or reference).

The appropriateness of using a MWU test in the context of differential methylation analysis is questionable, since the null hypothesis is difficult to precisely articulate except for the case of distributions that differ only by a shift in location [2]. Since the support of the probability distributions of the two estimators $\widehat{p}_n^{(t)}$ and $\widehat{p}_n^{(r)}$ is the unit interval, it is not always the case that these distributions differ only by a shift. This means that the MWU test will reject the underlying null hypothesis for differences other than location shifts. In this case, "interpretation of a small p-value is not always straightforward" [2]. This issue is further exacerbated by the failure of metilene to account for the data generation process that produces the $\widehat{p}$ values.

A simulation example demonstrates that the previous statistical framework is problematic. Let us consider the simplest possible context for methylation analysis: a CpG island (CGI) with 100 CpG sites that are methylated in an independent and identically distributed fashion with probability 0.05 in both the test and the reference samples. In this case, there is no differential methylation present within the CGI, since both the test and the reference samples are characterized by the same distributions for DNA methylation. However, let us assume that there is a difference in sequencing coverage between the test and reference samples, a situation that is common in real data. Recall that any valid statistical hypothesis testing procedure must control the Type I error rate (i.e., the percentage of false positives when the null hypothesis is true) at a level α when the null hypothesis is rejected for p-values less than α. Otherwise, the method cannot be trusted when it rejects the null hypothesis, since this could be a false-positive with high probability.

Using Monte Carlo, we simulated the previous example with 5 reference and 5 test samples subject to a small difference in coverage, which is quite common in WGBS sequencing data: $15\times$ in the test data and $10\times$ in the reference data. For a p-value threshold of 0.05, the Type I error rate of metilene was 16% (estimated from 10,000 Monte Carlo trials), more than three times over the maximum allowed in a correct statistical test. Specifically, if the statistical procedure were valid, the Type I error rate should have been no more than 5%. A higher difference in coverage worsens the problem. For instance, for a $30\times$ versus $10\times$ coverage, which is not outside the norm for WGBS data, metilene falsely calls this region as differentially methylated 98% of the time. Note that our simple example does not even consider additional complications of real-world data that would cause further problems for metilene: coverages that vary from CpG to CpG site within a given sample, missing data at certain CpG sites (although an attempt was made in [4] to fill-in missing data), correlation in methylation between CpG sites, etc. Notably, the issue of correlation results in violation of a critical assumption (independence) underlying the MWU test.

8.3 Differential analysis using the KS test

In addition to the MWU test, metilene employs another way to assess statistical significance using a two-dimensional version of the KS test. This test plays a crucial role in the circular binary segmentation algorithm used in the first mode of metilene. In addition, the KS test is used in the second mode to test for significant differences in each region of interest. However, no documentation has been provided on why a second statistical test is necessary, or why the KS test should be more preferable than the MWU test. The two tests are based on different formulations for the null and alternative hypotheses, and the lack of formal justification for either hypothesis testing procedure further contributes to the confusion. Regardless, the KS test cannot overcome the fundamental issue of ignoring the data generation process and the associated coverage information necessary to conduct valid statistical analysis. We demonstrate this fact next.

Metiline employs the KS test only in regions with multiple CpG sites. It is based on randomly selecting a CpG site within the region and calculating the associated maximum likelihood estimates $\widehat{p}^{(t)}$ and $\widehat{p}^{(r)}$ of the methylation probabilities in the test and reference samples, respectively. This process generates data points randomly distributed within a two-dimensional space determined by the two-dimensional random variable $(X^{(t)}, \widehat{p}^{(t)})$, where $X^{(t)}$ is a random variable indicating the location of the chosen CpG site. Likewise, the reference samples contribute data points randomly distributed within a two-dimensional space determined by the two-dimensional random variable $(X^{(r)}, \widehat{p}^{(r)})$. The two groups of points are then passed to a two-dimensional KS test to determine whether there is a statistically significant difference between the joint probability distributions of $(X^{(t)}, \widehat{p}^{(t)})$ and $(X^{(r)}, \widehat{p}^{(r)})$.

The issue of coverage variability seriously affects the KS test as well. Consider the simple case of $15\times$ test versus $10\times$ reference coverage. The maximum likelihood estimator $\widehat{p}_n^{(t)}$ at the n-th CpG site of the probability of methylation in the test sample distributes its probability mass over the set $\{0, 1/10, 2/10, \ldots, 1\}$, whereas the $\widehat{p}_n^{(r)}$ distributes its probability mass over a different set $\{0, 1/15, 2/15, \ldots, 1\}$. Therefore, even if the marginal probabilities of methylation were identical in the test and reference samples, one would expect the null hypothesis to be

rejected when there are different coverages between the samples. This is because the KS test is based on the null hypothesis that the cumulative probability distributions of $\widehat{p}_n^{(t)}$ and $\widehat{p}_n^{(r)}$ are identical. Indeed, by using the Monte Carlo simulation discussed in the previous subsection, we calculated a 100% false-positive rate for the case of 15× versus 10× coverage using 10,000 Monte Carlo samples, but worse-yet, even with a coverage of 11× versus 10×, the KS test still produces a 100% false positive rate.

There are additional problems associated with metilene's KS formulation. For example, by considering the random variable X, the null hypothesis of the KS test is false whenever the two samples have uneven missing data patterns within a region. To see why this is true, consider a case in which there are no missing data at the CpG sites in the test samples, while there are missing data in the reference samples. In this case, the probability distribution of $X^{(t)}$ in the test samples will follow a uniform distribution over the CpG locations within the region, while the probability distribution of $X^{(r)}$ in the reference samples will not be uniform. Therefore, the joint distribution of $(X^{(t)}, \widehat{p}^{(t)})$ will differ from the joint distribution of $(X^{(r)}, \widehat{p}^{(r)})$ regardless of the distributions of the methylation probabilities $\widehat{p}^{(t)}$ and $\widehat{p}^{(r)}$ in the region. Although metilene seems to handle missing data in a per CpG site basis, it can be the case that the set of CpG sites from each group included in the analysis is different for a given region of interest. This will happen when the total number of CpG sites in the region is above a certain threshold and when there is no data for a given CpG site in either group.

Clearly using the KS tests for determining regions of significant differential methylation between test and reference samples is highly problematic. As a result of our previous discussion and simple analysis, we conclude that metilene should not be used in practice. Only methods, such as DSS and informME, that properly account for the process that generates the sequencing data should be considered to be statistically valid procedures for differential methylation analysis.

9 bedGraph files generated by informME

Below we provide a list of the browser extensible data graph (bedGraph) tracks and excel spreadsheets generated by informME. The bedGraph files can be directly displayed in the UCSC genome browser (https://genome.ucsc.edu). "PhenoName" is the name for the phenotype used in inter-sample analysis (e.g., lungnormal-1). "tPhenoName" and "rPhenoName" are the names of the test and reference phenotypes used in differential analysis (e.g., lungcancer-1 and lungnormal-1, respectively). Finally, "tName" and "rName" are names for the test and reference phenotypes used in differential analysis (e.g., lungcancer and lungnormal, respectively).

Single-Sample Statistics

- **MML-PhenoName.bed**: mean methylation levels within GUs
- **NME-PhenoName.bed**: normalized methylation entropies within GUs

Single-Sample Classification

- **METH-PhenoName.bed**: methylation-based classifications of GUs (non-variable)
  - −2: highly unmethylated
  - −1: partially unmethylated
  - 0: variably methylated
  - 1: partially methylated
  - 2: highly methylated
- **VAR-PhenoName.bed**: methylation-based classifications of GUs (variable)
  - 1: mixed
  - 2: highly mixed
  - 3: bistable
- **ENTR-PhenoName.bed**: entropy-based classifications of GUs
  - −2: highly ordered
  - −1: moderately ordered
  - 0: weakly ordered/disordered
  - 1: moderately disordered
  - 2: highly disordered

Differential Statistics

- **dMML-tPhenoName-VS-rPhenoName.bed**: differential mean methylation level statistics within GUs
- **dNME-tPhenoName-VS-rPhenoName.bed**: differential normalized methylation entropy statistics within GUs
- **JSD-tPhenoName-VS-rPhenoName.bed**: Jensen-Shannon distance statistics within GUs

Differential Classification

- **DMU-tPhenoName-VS-rPhenoName.bed**: differential methylation-based classifications of GUs
  - −3: test is strongly hypomethylated
  - −2: test is moderately hypomethylated
  - −1: test is weakly hypomethylated
  - 0: isomethylated
  - 1: test is weakly hypermethylated
  - 2: test is moderately hypermethylated
  - 3: test epigenotype is strongly hypermethylated

- **DEU-tPhenoName-VS-rPhenoName.bed**: differential entropy-based classifications of GUs

  -3: test is strongly hypoentropic
  -2: test is moderately hypoentropic
  -1: test is weakly hypoentropic
  0: isoentropic
  1: test is weakly hyperentropic
  2: test is moderately hyperentropic
  3: test is strongly hyperentropic

Differential Regions

- **DMR-JSD-tPhenoName-VS-rPhenoName.bed**: differentially methylated regions with statistical scores.

Gene Ranking

- **gRank-JSD-tName-VS-rName.xlsx**: Excel spreadsheet containing a list of ranked genes using the JSD when no replicate reference data is available.
- **gRankRDD-JSD-rName-VS-rName.xlsx**: Excel spreadsheet containing a list of ranked genes using the JSD when replicate reference data is available.

References

1. P. J. Bickel and K. A. Doksum. *Mathematical Statistics: Basic Ideas and Selected Topics*, volume I. Prentice-Hall, Upper Saddle River, New Jersey, 2nd edition, 2007.
2. M. W. Fagerland. t-tests, non-parametric tests, and large studies - a paradox of statistical practice? *BMC Med Res Methodol*, 12:78, 2012.
3. G. Fasano and A. Franceschini. A multidimensional version of the Kolmogorov-Smirnov test. *Mon Not R Astr Soc*, 255:155–170, 1987.
4. F. Jühling, H. Kretzmer, S. H. Bernhart, C. Otto, P. F. Stadler, and S. Hoffmann. metilene: fast and sensitive calling of differentially methylated regions from bisulfite sequencing data. *Genome Res*, 26:256–262, 2016.
5. A. Mohammad-Djafari. A Matlab program to calculate the maximum entropy distributions. In C R Smith, G Erickson, and P O Neudorfer, editors, *Maximum Entropy and Bayesian Methods*, volume 50 of *Fundamental Theories of Physics*, pages 221–233. Springer, New York, 1992.
6. S. Pressé, K. Ghosh, J. Lee, and K. A. Dill. Principles of maximum entropy and maximum caliber in statistical physics. *Rev Mod Phys*, 85:1115–1141, 2013.

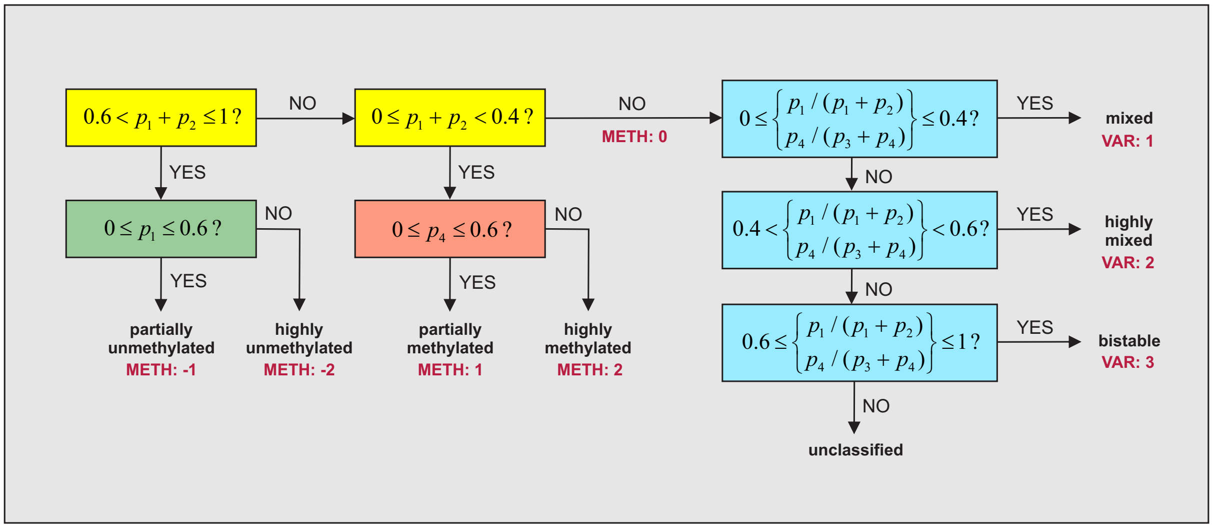

Figure S1. Methylation-based classification scheme for GUs that leads to two genome-wide bedGraph tracks: METH and VAR (see Section 9 for the associated codes). The probabilities p_1, p_2, p_3, and p_4 are given by (S23). Note that a (small) number of GUs are not classified by this scheme.

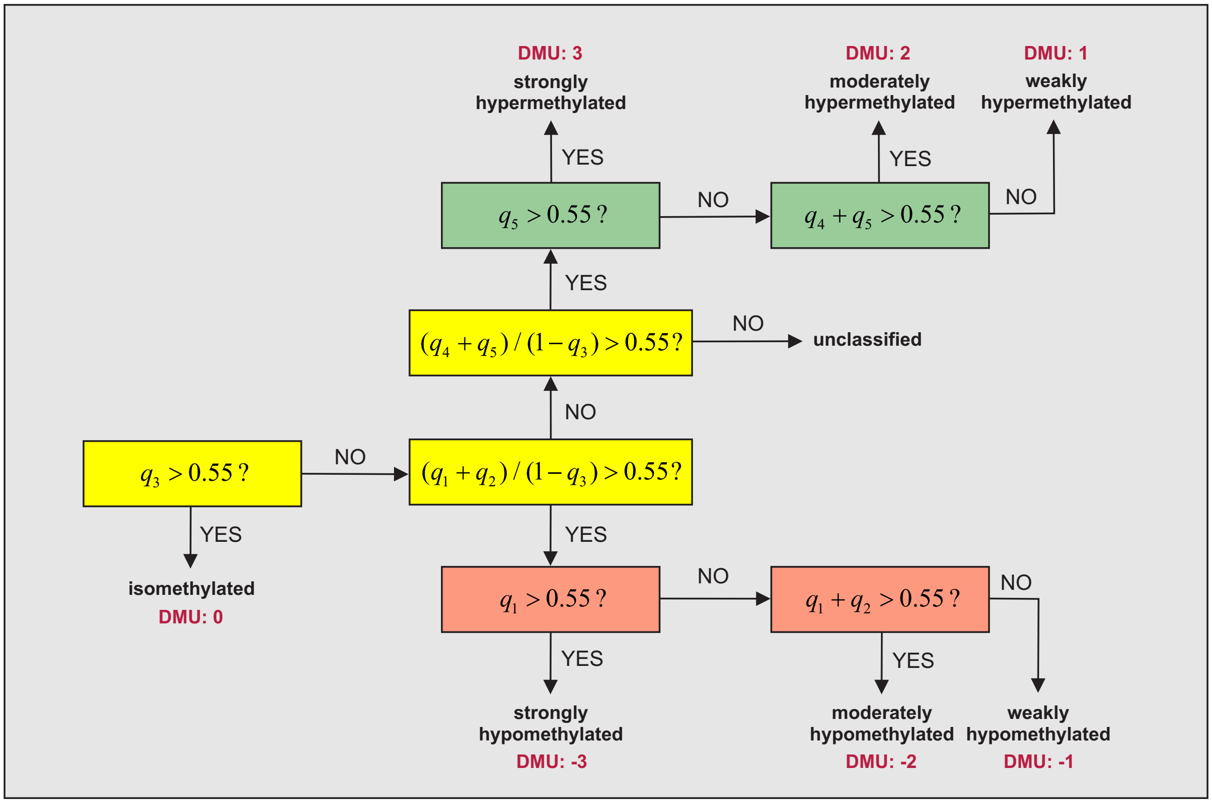

Figure S2. Methylation-based differential classification scheme for GUs that leads to the genome-wide DMU bedGraph track (see Section 9 for the associated codes). The probabilities q_1, q_2, q_3, q_4, and q_5 are given by (S25). Note that a (small) number of GUs are not classified by this scheme.

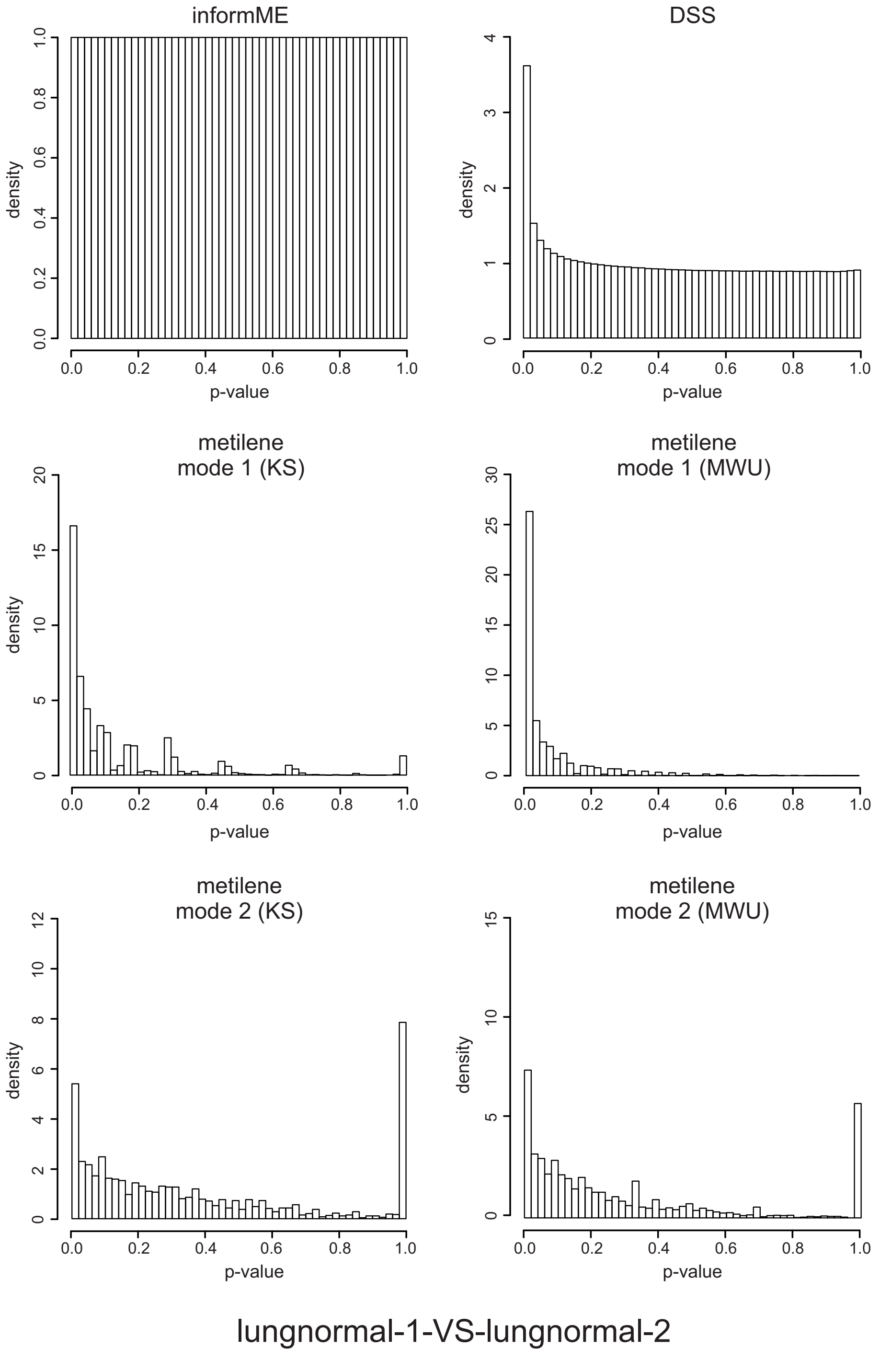

Figure S3. Distribution of p-values obtained genomewide using the (lungnormal-1, lungnormal-2) pair of our lung normal data by informME, DSS-single, metilene in the "DMR de-novo annotation" mode 1 based on the KS test statistic, metilene in the "DMR de-novo annotation" mode 1 based on the MWU test statistic, metilene in "DMR annotation in known features" mode 2 based on the KS test statistic, and metilene in "DMR annotation in known features" mode 2 based on the MWU test statistic.

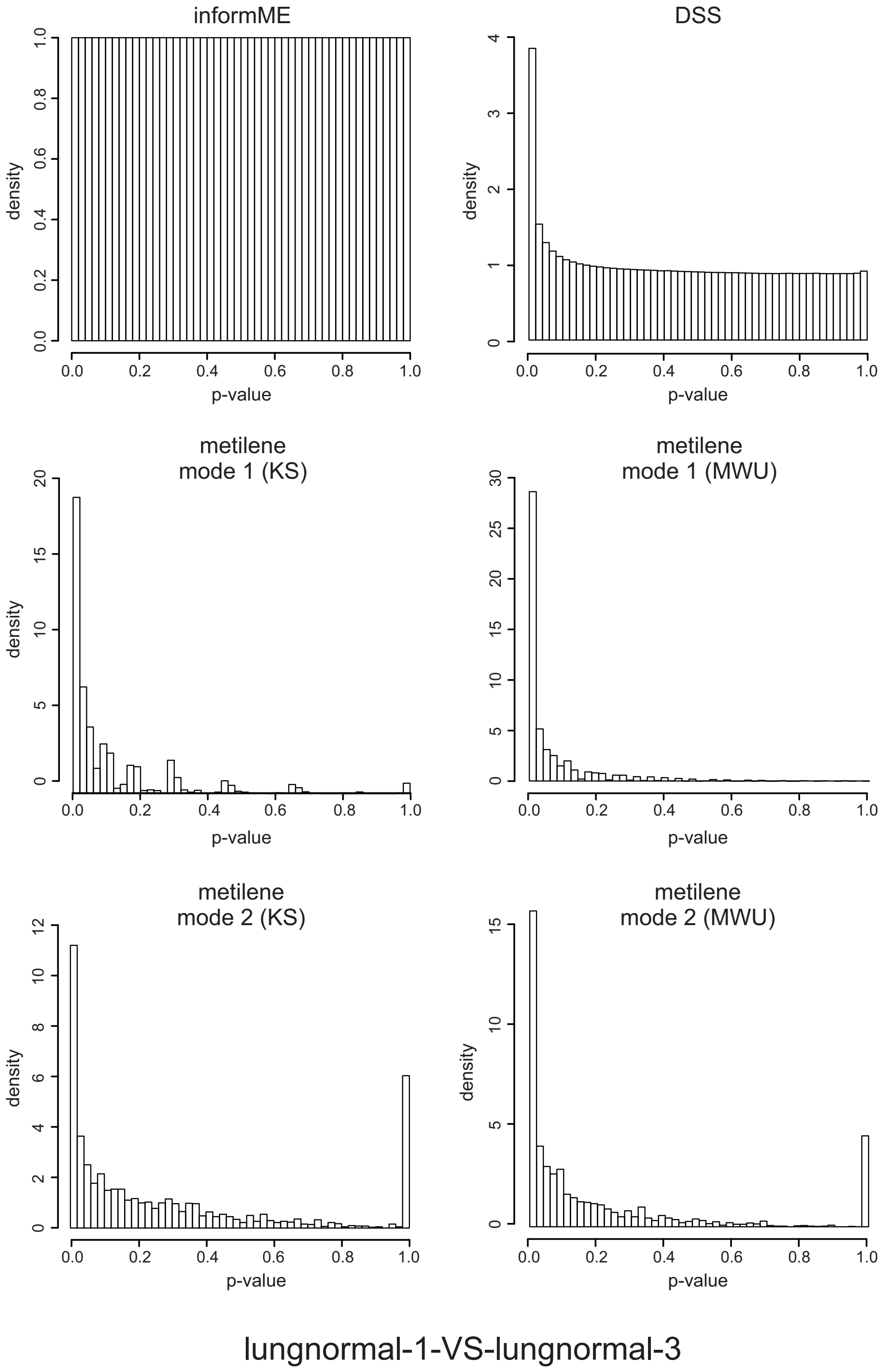

Figure S4. Distribution of p-values obtained genomewide using the (lungnormal-1, lungnormal-3) pair of our lung normal data by informME, DSS-single, metilene in the "DMR de-novo annotation" mode 1 based on the KS test statistic, metilene in the "DMR de-novo annotation" mode 1 based on the MWU test statistic, metilene in "DMR annotation in known features" mode 2 based on the KS test statistic, and metilene in "DMR annotation in known features" mode 2 based on the MWU test statistic.

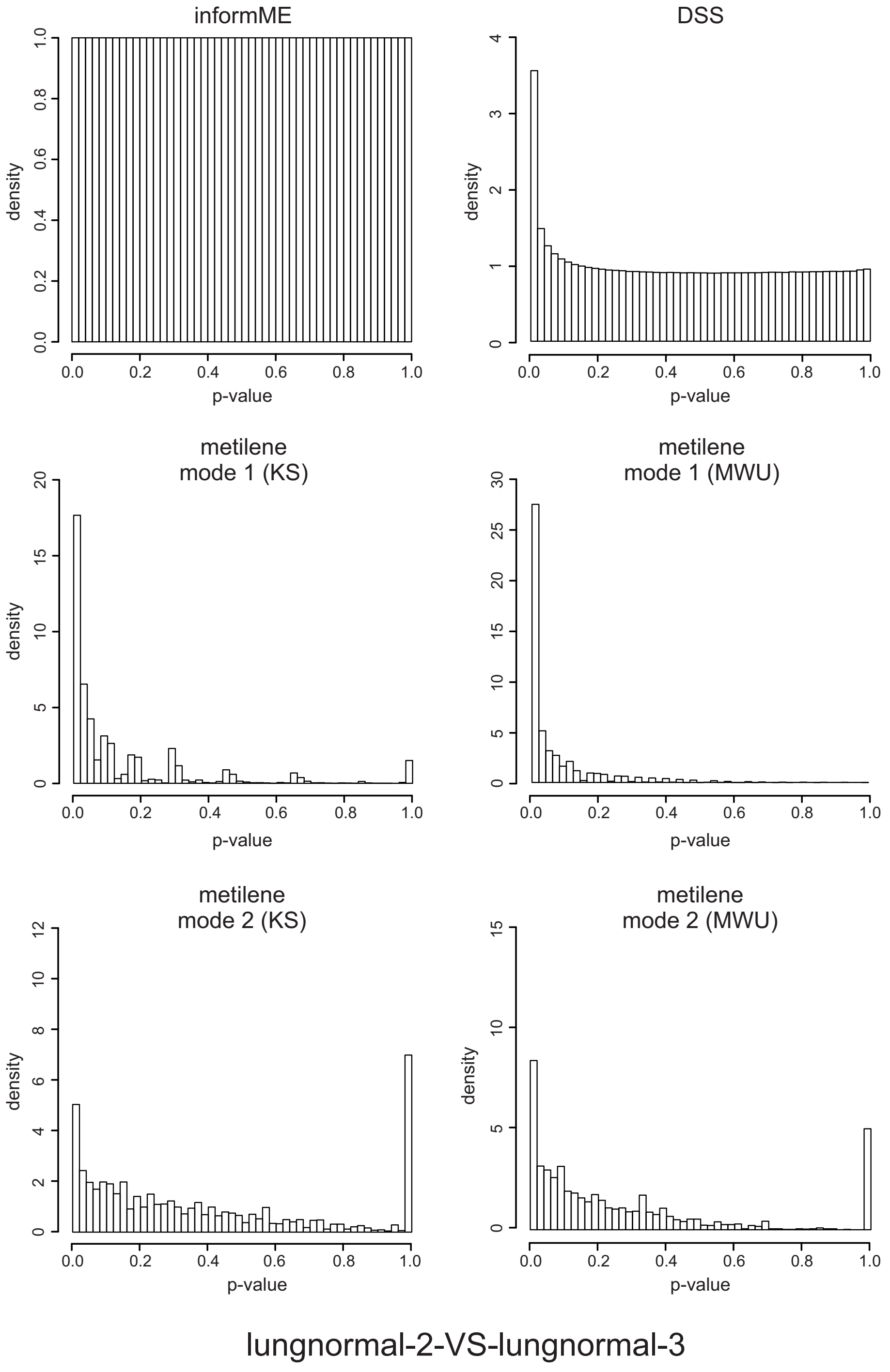

Figure S5. Distribution of p-values obtained genomewide using the (lungnormal-2, lungnormal-3) pair of our lung normal data by informME, DSS-single, metilene in the "DMR de-novo annotation" mode 1 based on the KS test statistic, metilene in the "DMR de-novo annotation" mode 1 based on the MWU test statistic, metilene in "DMR annotation in known features" mode 2 based on the KS test statistic, and metilene in "DMR annotation in known features" mode 2 based on the MWU test statistic.

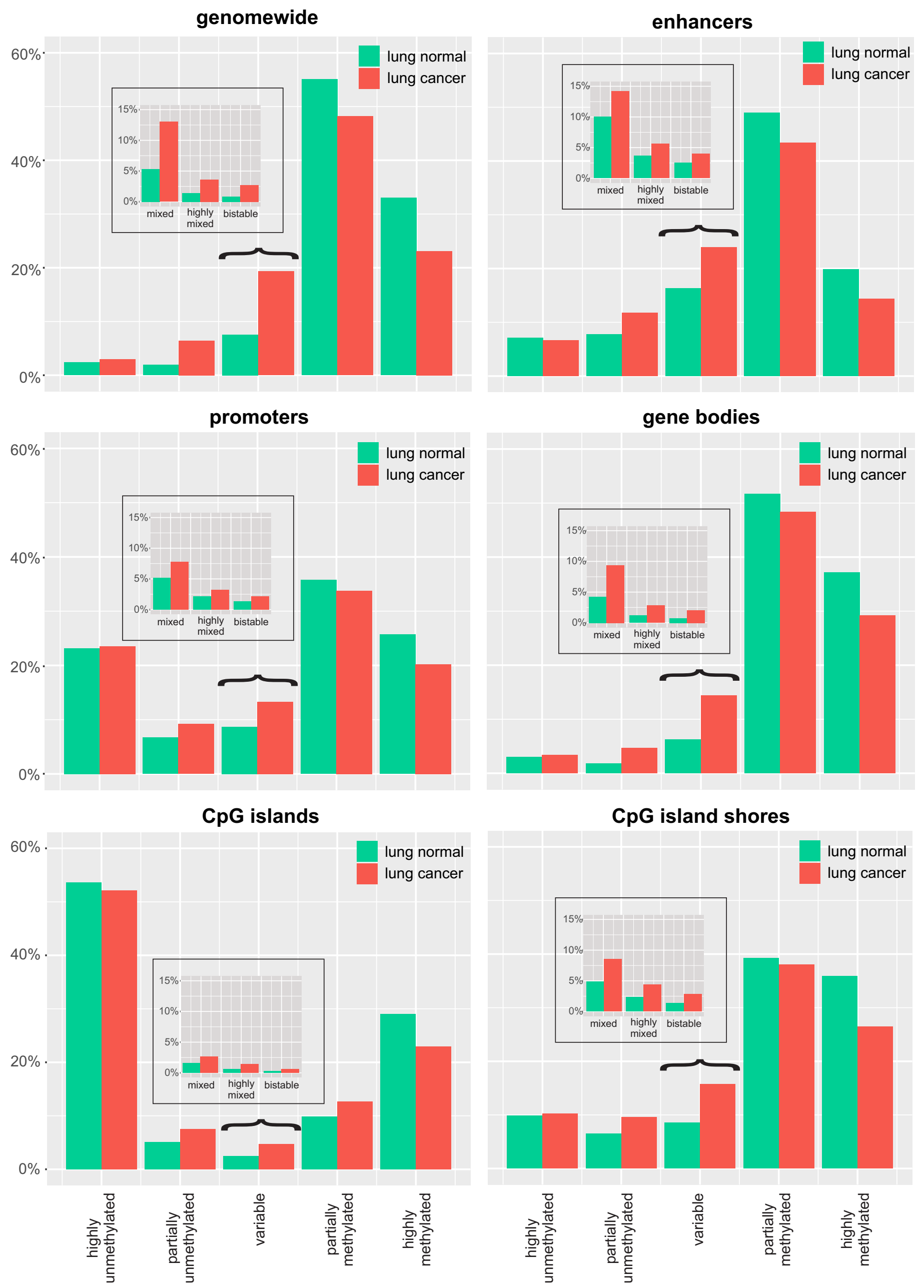

Figure S6. Distributions of aggregate methylation-based GU classifications (METH and VAR tracks – see Section 9) within the entire genome, enhancers, promoters, gene bodies, CGIs, and CGI shores.

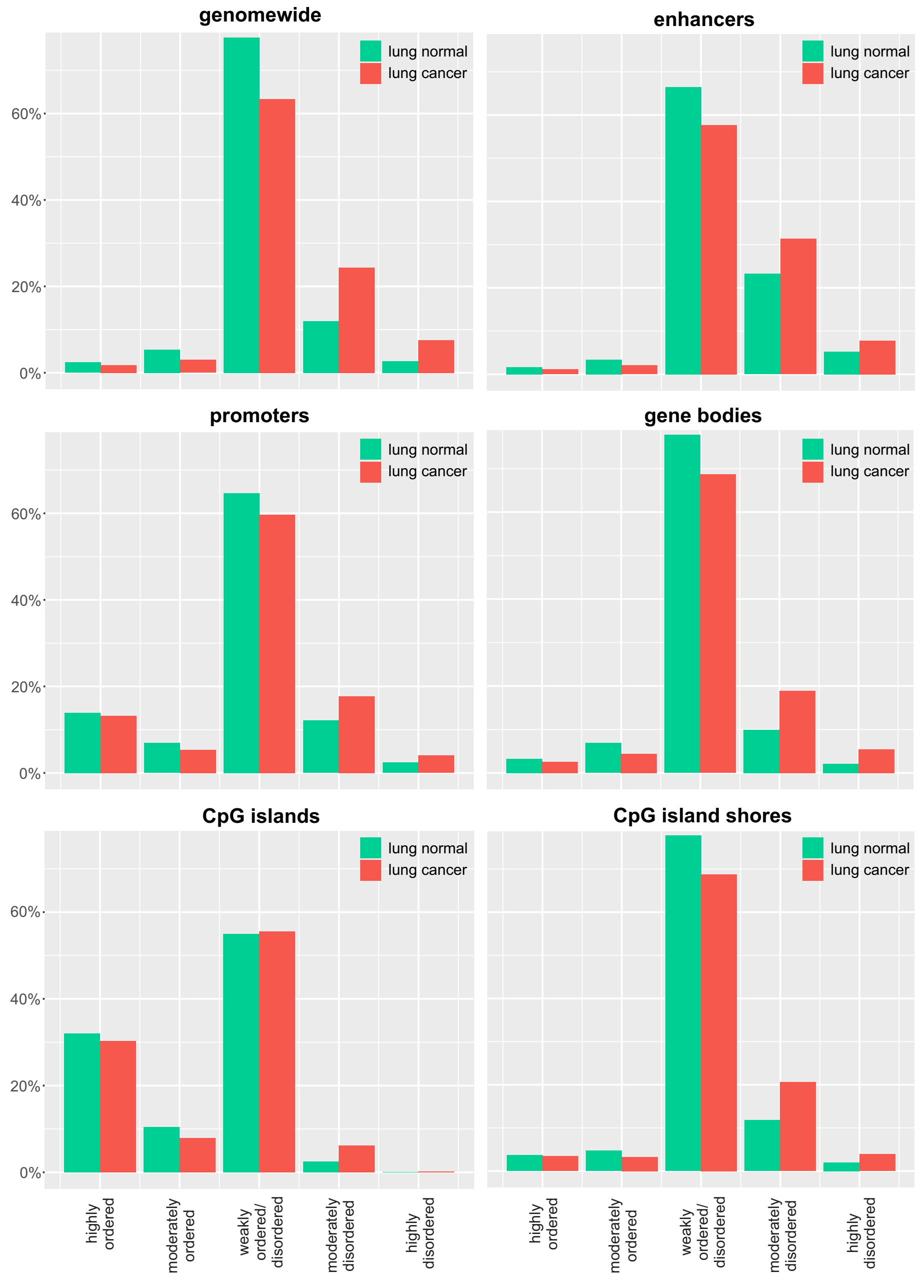

Figure S7. Distributions of aggregate entropy-based GU classifications (ENTR track – see Section 9) within the entire genome, enhancers, promoters, gene bodies, CGIs, and CGI shores.

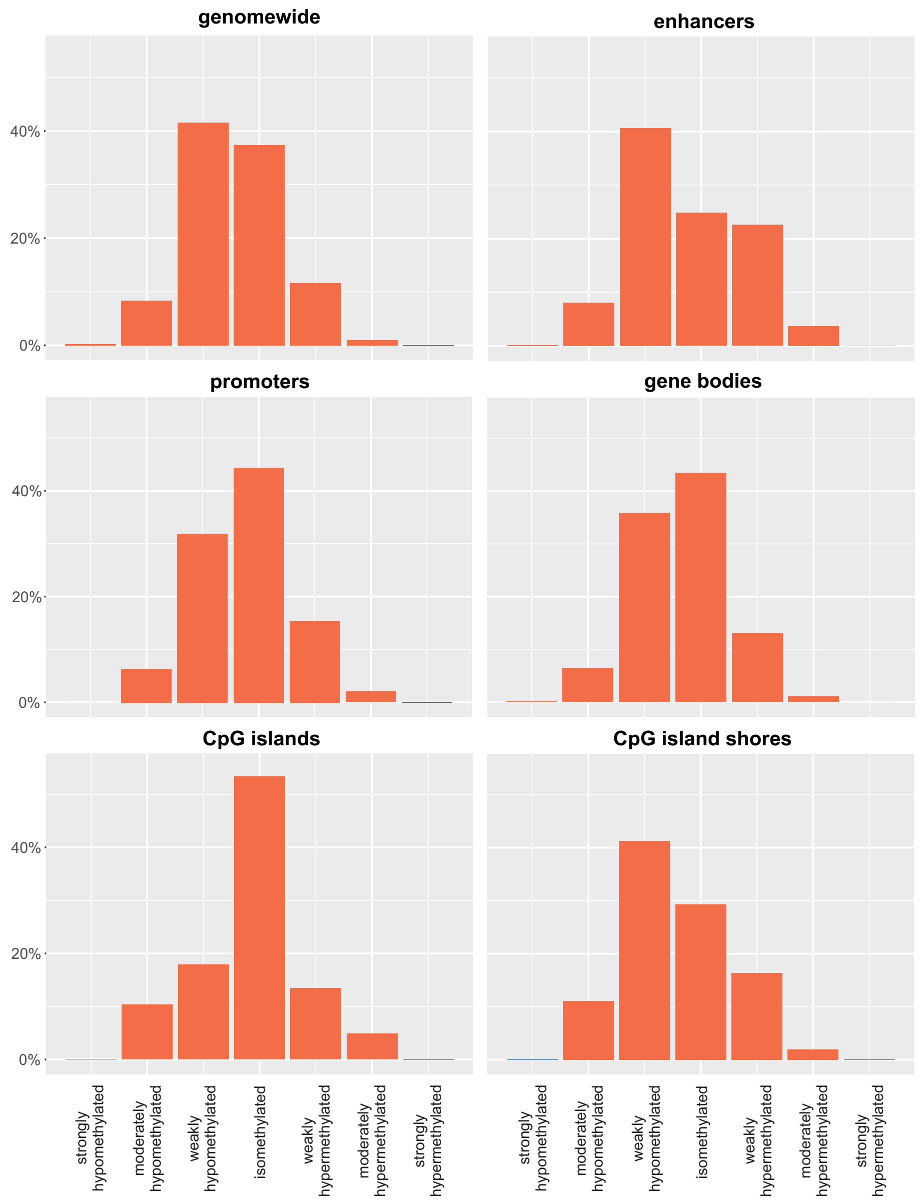

Figure S8. Distributions of aggregate methylation-based differential GU classifications (DMU track – see Section 9) within the entire genome, enhancers, promoters, gene bodies, CGIs, and CGI shores.

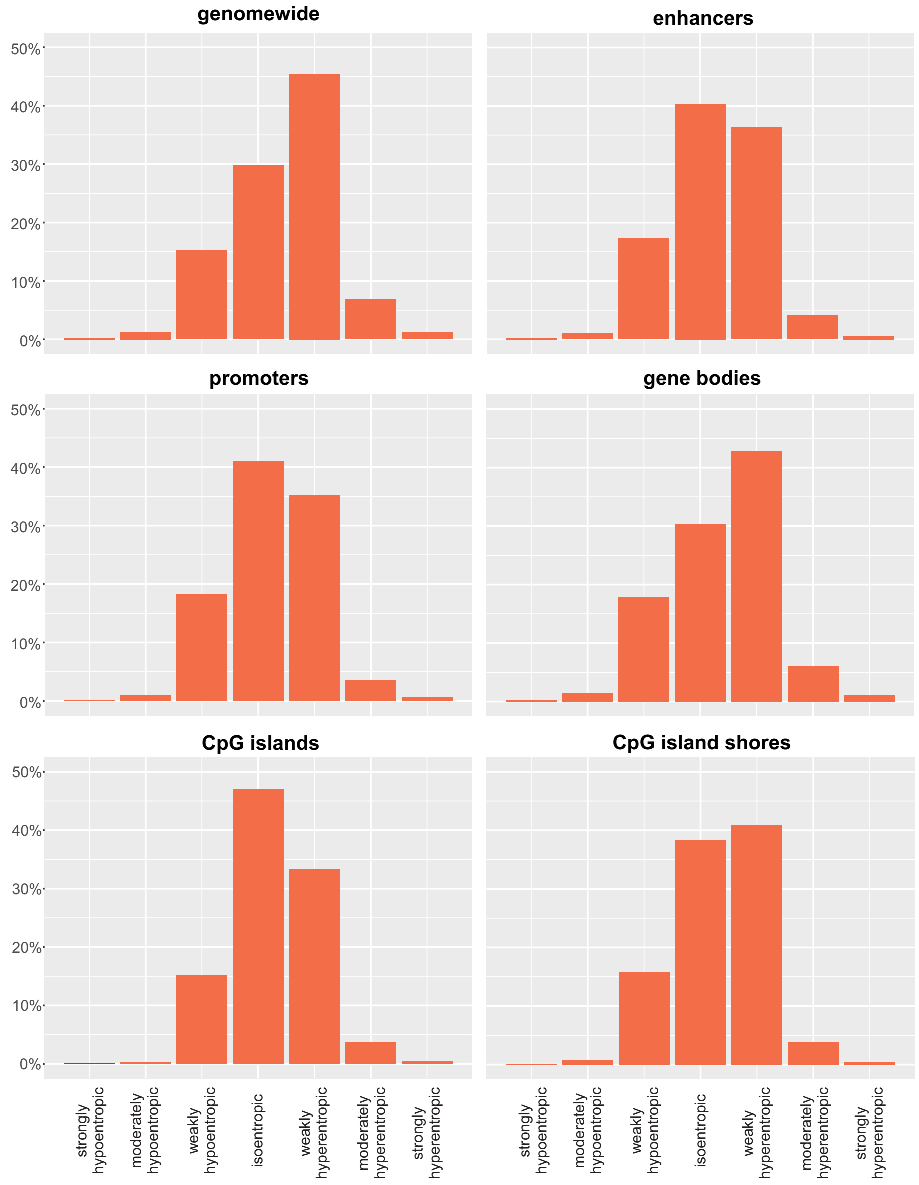

Figure S9. Distributions of aggregate entropy-based differential GU classifications (DEU track – see Section 9) within the entire genome, enhancers, promoters, gene bodies, CGIs, and CGI shores.

Table S1. CPU time and maximum RAM requirements for informME and DSS when applied on our (lungcancer-3, lungnormal-3) pair of samples.

METHOD	TASK	CPU time (h)	max RAM (Gb)
**informME**	Model Estimation	1,647	9
	Inter-Sample Methylation Analysis	246	8
	Differential Methylation Analysis	4.8	7
	DMR Detection	0.2	3
	**Total**	**1,898**	**9**
**DSS**	Model Estimation	233	23
	DMR Detection	59	40
	**Total**	**292**	**40**
